# Lysophosphatidylglycerol (LPG) phospholipase D maintains membrane homeostasis in *Staphylococcus aureus* by converting LPG to lysophosphatidic acid

**DOI:** 10.1016/j.jbc.2023.104863

**Published:** 2023-05-25

**Authors:** Chitra Subramanian, Mi-Kyung Yun, Matthew M. Frank, Charles O. Rock

**Affiliations:** Department of Infectious Diseases, St Jude Children’s Research Hospital, Memphis, Tennessee, USA

**Keywords:** Staphylococus aureus, phosphatidylglycerol, lysophosphtidylglycerol, membrane, phospholipase D, phospholipid

## Abstract

Lysophospholipids are deacylated derivatives of their bilayer forming phospholipid counterparts that are present at low concentrations in cells. Phosphatidylglycerol (PG) is the principal membrane phospholipid in *Staphylococcus aureus* and lysophosphatidylglycerol (LPG) is detected in low abundance. Here, we used a mass spectrometry screen to identify locus *SAUSA300_1020* as the gene responsible for maintaining low concentrations of 1-acyl-LPG in *S. aureus*. The *SAUSA300_1020* gene encodes a protein with a predicted amino terminal transmembrane α-helix attached to a globular glycerophosphodiester phosphodiesterase (GDPD) domain. We determined that the purified protein lacking the hydrophobic helix (LpgDΔN) possesses cation-dependent lysophosphatidylglycerol phospholipase D activity that generates both lysophosphatidic acid (LPA) and cyclic-LPA products and hydrolyzes cyclic-LPA to LPA. Mn^2+^ was the highest affinity cation and stabilized LpgDΔN to thermal denaturation. LpgDΔN was not specific for the phospholipid headgroup and degraded 1-acyl-LPG, but not 2-acyl-LPG. Furthermore, a 2.1 Å crystal structure shows that LpgDΔN adopts the GDPD variation of the TIM barrel architecture except for the length and positioning of helix α6 and sheet β7. These alterations create a hydrophobic diffusion path for LPG to access the active site. The LpgD active site has the canonical GDPD metal binding and catalytic residues, and our biochemical characterization of site-directed mutants support a two-step mechanism involving a cyclic-LPA intermediate. Thus, the physiological function of LpgD in *S. aureus* is to convert LPG to LPA, which is re-cycled into the PG biosynthetic pathway at the LPA acyltransferase step to maintain membrane PG molecular species homeostasis.

Phospholipids are the building blocks used to construct membrane bilayers (1), and their deacylated forms that carry a single acyl chain are called lysophospholipids. The solubility of lysophospholipids means that they can be secreted from cells to carry out signaling functions (2, 3), and they participate in deacylation-reacylation cycles in mammals that remodel the membrane phospholipid molecular species composition (4). Consistent with these roles in biology, lysophospholipids are normally present in low abundance (<1%) relative to bilayer forming phospholipids in most cells, although their concentrations may rise in certain pathophysiological states (5). The accumulation of high concentrations of lysophospholipids destabilizes the membrane bilayer, and active metabolic processes exist to maintain their concentrations at low levels relative to their bilayer forming phospholipid counterparts (2). In bacteria, a signaling function for lysophospholipids has not been discovered and there is no evidence for deacylation/reacylation cycle that re-organizes the phospholipid molecular species composition. Rather, lysophospholipids arise in bacteria from the degradation of membrane phospholipids by endogenous (6) or exogenous (7) phospholipases, or from the use of phospholipids as acyl donors in the biosynthesis of other molecules (5). A well-studied example is the use of phosphatidylethanolamine, the major phospholipid of *Escherichia coli*, as a substrate for phospholipid:apolipoprotein transacylase (Lnt) (8, 9). Lnt catalyzes the transfer of the acyl chain at the 1-position of phosphatidylethanolamine to the lipoprotein amino terminus. The resulting 2-acyl-*sn*-glycero-3-phosphoethanolamine (2-acyl-LPE) is transported into the cell interior by LplT (10) and the 1-position is acylated by the acyl-acyl carrier protein dependent 2-acyl-LPE acyltransferase (11, 12). *Staphylococcus aureus* also uses membrane phospholipid as substrate in the amino-terminal acylation of lipoproteins by LnsAB, a heterodimeric phospholipid:apolipoprotein transacylase with the subunits derived from separate genes (13). However, there are no orphan acyltransferases in *S. aureus* and the metabolic fate of lysophospholipids is not known in this pathogen.

Phosphatidylglycerol (PG) is the major membrane phospholipid of *S. aureus* and consists of molecular species containing ≥16-carbon acyl chains in the 1-position and 12(S)-methyltetradecanoic acid (a15) esterified at the 2-position (14, 15). Lysophosphatidylglycerols (LPG) are present at low concentrations. The major LPG species is a15-LPG arising from the phospholipase A1 activity of glycerol ester hydrolase (16). The bulk of the a15-LPG is released into the media, and the 1-position fatty acids removed from PG in the synthesis of a15-LPG are recycled into the phospholipid biosynthetic pathway at the PlsY step following their activation by a fatty acid kinase (17). The other LPG molecular species consist of ≥16-carbon acyl chains (≥16-LPG) that arise from the removal of a15 from the 2-position (16). The metabolic process(es) creates the ≥16-LPG pool is unknown.

This study identifies a new enzyme in bacterial lipid metabolism, lysophosphatidylglycerol phospholipase D (LpgD) that is responsible for maintaining a low concentration of 1-acyl-LPG in the *S. aureus* membrane. The *lpgD* gene was identified by a targeted mutant screen, and the purified protein exhibited cation-dependent phosphodiesterase activity converting LPG to a cyclic-lysophosphatidic acid (cLPA) intermediate that is hydrolyzed to lysophosphatidic acid (LPA). The 2.1 Å crystal structure shows LpgD adopts the canonical glycerophosphodiester phosphodiesterase (GDPD) TIM barrel fold except for the positioning and length of helix α6 and sheet β7 that create a hydrophobic docking platform for the LPG acyl chain. LgpD has the canonical active site residues found in the GPDP protein family and site-directed mutagenesis establishes the contributions of the active site histidines (His51 and His93) and the Mn^2+^ center in the formation and hydrolysis of the cLPA intermediate. Thus, members of the GlpQ family of phosphodiesterases operate *via* a cyclic-phosphate rather than a phospho-histidine intermediate. The specific physiological function of LpgD in *S. aureus* is to re-cycle LPG into the PG biosynthetic pathway at the LPA acyltransferase step to maintain membrane PG molecular species homeostasis.

## Result

### Identification of the lpgD gene

The goal was to identify the gene responsible for maintaining the low concentration of ≥16-LPG in *S. aureus*. There are no orphan acyltransferase genes in *S. aureus*; therefore, we screened mutants in predicted α/β hydrolases (lipases) and phosphodiesterases that may potentially hydrolyze ≥16-LPG to prevent its accumulation in the membrane. We selected strains from the Nebraska transposon library (18) containing inactivating mariner transposon (φNΣ) insertions in each of these candidate genes (Table S1) and screened them using LC-MS/MS to determine if any of these deletion strains have altered cellular concentrations of ≥16-LPG compared to the wild-type parent, strain JE2 (Fig. S1*A*). In this screen, we measured the relative abundance of *anteiso*-17-LPG (a17-LPG), the most abundant of the ≥16-LPG species present in *S. aureus* (16), using [d5]17-LPG as the internal standard. None of the strains harboring inactivating insertions in the α/β hydrolase/lipase/phospholipase genes had altered cellular concentrations of ≥16-LPG. However, strain NE1544 harboring a φNΣ insertion in the *SAUSA300_1020* gene exhibited a clear, ∼15-fold increase in the cellular content of a17-LPG compared to the parent strain JE2 (Fig. S1*A*). The *SAUSA300_1020* gene is bioinformatically related to the GlpQ family of GDPD phosphodiesterases. We named the *SAUSA300_1020* gene *lpgD* (lysophosphatidylglycerol phospholipase D) based on the experimental results described in the following sections.

**Figure 1:**
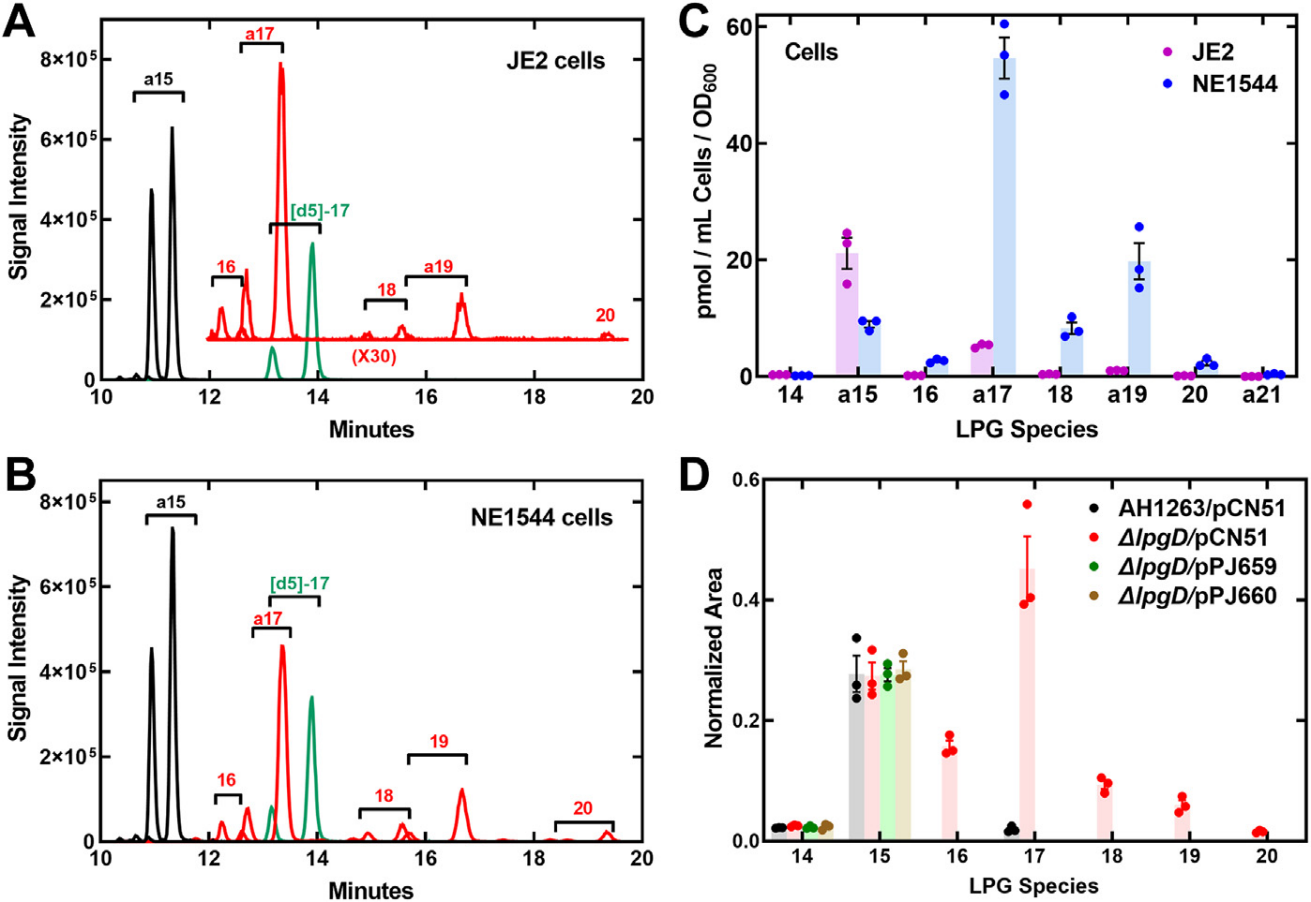
**Identification of the *lpgD* gene.***A*, A representative LC-MS/MS scan illustrating the LPG molecular species of strain JE2 (wild-type) with the [d5]17-LPG internal standard (*green*), a15-LPG (*black*), and ≥16-LPG (*red*). *Inset*, the signal channels for ≥16-LPG are amplified by 30x to clearly visualize the LPG molecular species composition. *B*, A representative LC-MS/MS scan illustrating the elevated concentrations of LPG molecular species in strain NE1544 (*lpgD*::φNΣ). There is no amplification of the ≥16-LPG signals in this panel. *C*, quantitation of the cellular concentrations of LPG molecular species in strains JE2 (wild-type) and NE1544 (*lpgD*::φNΣ) determined by LC-MS/MS using [d5]17-LPG as the internal standard. *D*, complementation of strain PDJ73 (Δ*lpgD*) with plasmids expression either LpgD (pPJ659) or LpgDΔN (pPJ660). Strain AH1263 is the parent strain of strain PDJ73 and harbors the empty vector, pCN51. The relative areas of each LPG molecular species were measured by LC-MS/MS with respect to [d5]17-LPG as the internal standard.

The size and molecular species composition of the LPG pool in strain NE1544 (*lpgD*::φNΣ) was compared to the parent strain JE2. Strain JE2 had detectable amounts of ≥16-LPG, but a15-LPG was the predominant component of the LPG pool (16) (Fig. 1*A*). Strain NE1544 (*lpgD*::φNΣ) had comparable amounts of a15-LPG coupled with greatly elevated amounts of ≥16-LPG (Fig. 1*B*). Although the total amount of ≥16-LPG increased in the *lpgD*::φNΣ strain, the composition of the ≥16-LPG molecular species did not change (Fig. 1*C*). The cellular concentrations of a15-LPG were slightly lower in strain NE1544 illustrating that the *lpgD gene* was only involved in the metabolism of ≥16-LPG. While a15-LPG is abundant in the media, the ≥16-LPG are in low abundance and difficult to detect (Fig. S2*A*) (16). However, in strain NE1544 (*lpgD*::φNΣ), the abundance of ≥16-LPG was significantly elevated in the media (Fig. S2, *B* and *C*). These data show that release of ≥16-LPG into the environment was a secondary mechanism used by *S. aureus* to cope with an elevated ≥16-LPG pool. However, the quantification of extracellular ≥16-LPG showed that the amount of ≥16-LPG detected in the media (Fig. S2*C*) was less than the amounts retained by the cells (Fig. 1*C*). The extracellular concentration of a15-LPG was unchanged in strain NE1544 compared to strain JE2 (Fig. S1*C*). These data show that ≥16-LPG metabolism is selectively impacted in strain NE1544 (*lpgD*::φNΣ).

Next, we prepared an unmarked deletion of the *lpgD* gene in wild-type *S. aureus* strain AH1263 to validate the results with the φNΣ insertion (Fig. S1, *B* and *C*). Strain PDJ73 (Δ*lpgD*) also had greatly elevated cellular levels of ≥16-LPG (Fig. 1*D*) confirming the metabolic phenotype of a Δ*lpgD* knockout strain. Plasmids that express either full-length LpgD or LpgD lacking the first 24 amino acids (LpgDΔN) were constructed in the Cd-inducible pCN51 expression vector (19), transformed into strain PDJ73, and the cellular levels of ≥16-LPG determined by LC-MS/MS (Fig. 1*D*). Strain PDJ73 (Δ*lpgD*)/pCN51 had a massive increase in ≥16-LPG compared to the parent strain AH1263/pCN51. The plasmid-driven expression of either LpgD (PDJ73 (Δ*lpgD*)/pPJ659) or LpgDΔN (PDJ73 (Δ*lpgD*)/pPJ660) restored the low concentrations of cellular ≥16-LPG characteristic of the wild-type strain (Fig. 1*D*). These data show that the *lpgD* gene is both necessary and sufficient for maintaining low ≥16-LPG concentrations in *S. aureus*. These data also show that when expressed using pCN51, the LpgDΔN protein lacking the first 24 amino acids of LpgD was also capable of normalizing the cellular amounts of ≥16-LPG.

### Biochemical properties of LpgDΔN

#### Purification of LpgDΔN

The *lpgD* gene is predicted to encode a protein with an amino-terminal, 24 amino acid α-helix attached to a globular GDPD domain found in the GlpQ family of bacterial phosphodiesterases (Fig. S3*A*). The amino terminal α-helix was interpreted as a membrane anchor based on its length and hydrophobicity, and the absence of bacterial signal sequence motifs in LpgD makes it highly unlikely to be a secretion signal (20). Therefore, recombinant protein (LpgDΔN) with a carboxy-terminal His-tag was expressed in *E. coli* and LpgDΔN was purified by Ni^2+^ affinity followed by gel filtration chromatography (Fig. S3*B*). Gel filtration chromatography indicated that LpgDΔN was a monomer based on analytical ultracentrifugation (Table S2 and Fig. S3*B*, *Inset*). LpgDΔN was pure as judged by SDS gel electrophoresis (Fig. S3*C*).

#### Cation requirement

**Figure 2:**
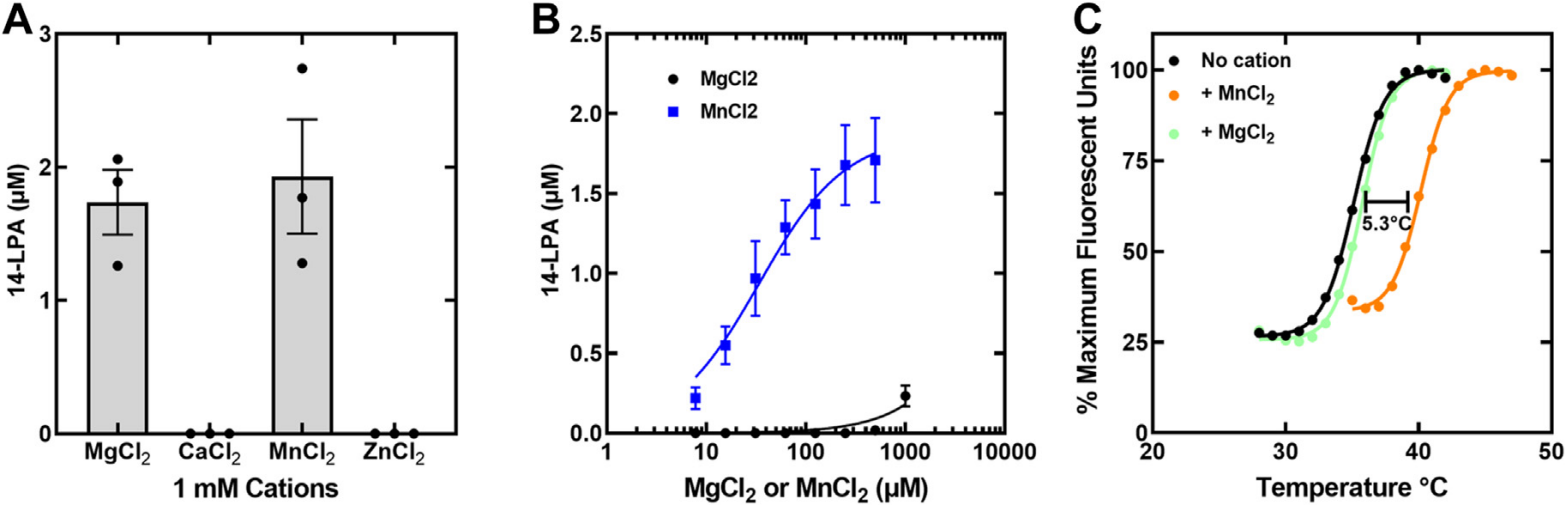
**LpgD cation preference**. *A*, LpdDΔN assays were performed with the indicated cation and the abundance of 14-LPA product determined by LC-MS/MS as described under Experimental procedures. *B*, the apparent K_M_ of LpgDΔN for Mn^2+^ (29.3 ± 4.9 μM) and Mg^2+^ (>1 mM) was determined using 14-LPG as substrate. *C*, the thermal stability of LpgDΔN in the absence and presence of Mg^2+^ or Mn^2+^.

The established function of the GDPD family of enzymes is to hydrolyze deacylated phospholipids liberating the phospholipid headgroup alcohol and *sn*-glycerol-3-phosphate (21, 22). Therefore, the predicted activity of LpgDΔN based on its function in cell physiology (Fig. 1) is the formation of lysophosphatidic acid (LPA) from LPG. GDPD enzymes have a divalent cation requirement, usually Mg^2+^, although there are examples of GDPD enzyme using Ca^2+^, Mn^2+^ or Zn^2+^ (22, 23). We screened LpgDΔN for activity using these four divalent cations using LC-MS/MS to detect LPA formation to be employed by GDPD family members (Fig. 2*A*). Both Mn^2+^ and Mg^2+^ supported LpgDΔN lysophospholipase D activity, whereas Ca^2+^ and Zn^2+^ did not. The apparent K_M_’s of LpgDΔN for Mn^2+^ and Mg^2+^ showed that Mn^2+^ was the most efficient cation in supporting LPA formation (Fig. 2*B*). Mn^2+^ also stabilized LpgDΔN to thermal denaturation by 5.3 °C, whereas the addition of Mg^2+^ had little impact on the stability of LpgDΔN (Fig. 2*C*). These data show that LpgDΔN has the highest affinity for Mn^2+^ but that Mg^2+^ also supports the reaction at higher concentrations.

#### Positional specificity

**Figure 3:**
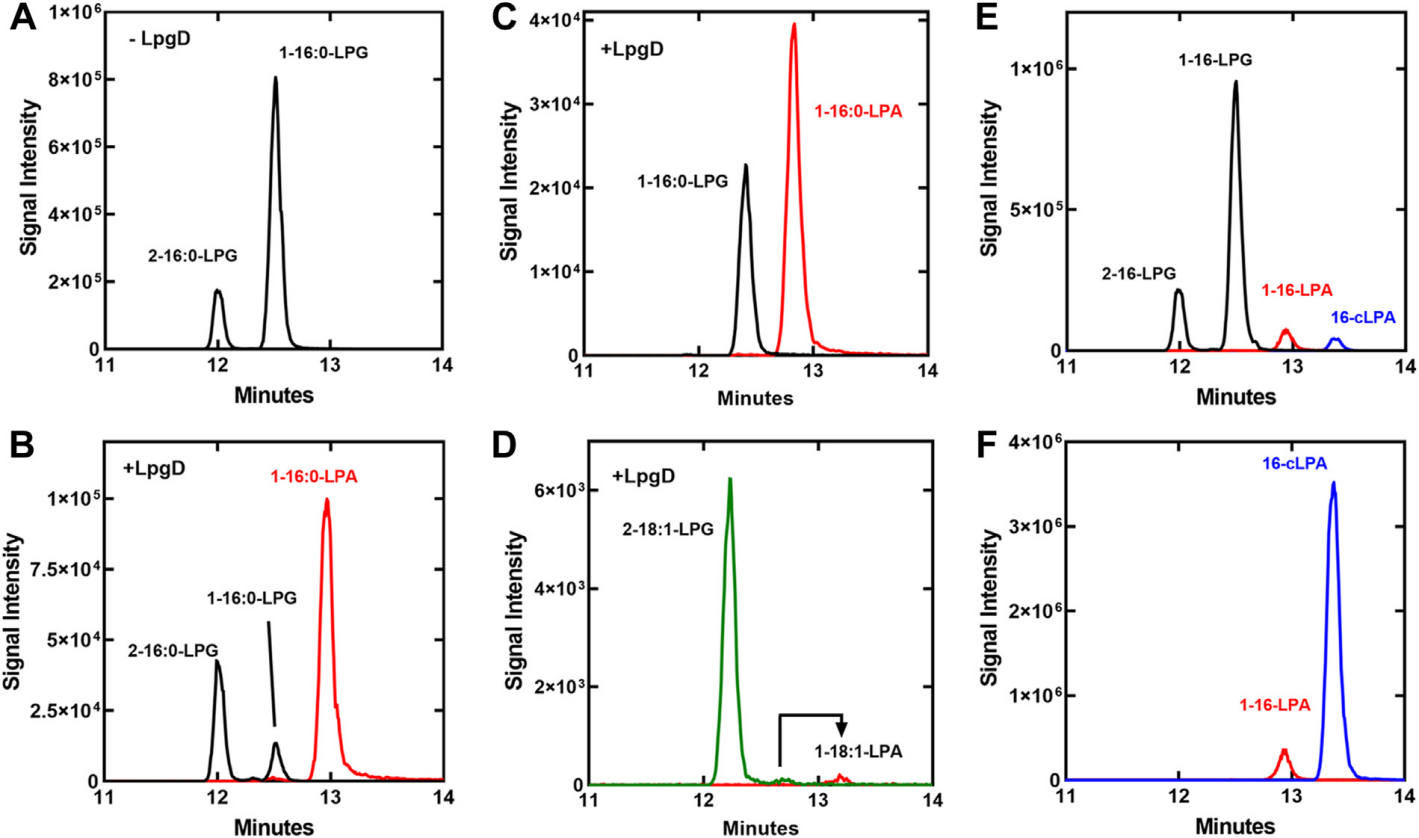
**Substrates and products of LpgDΔN**. LpgDΔN (250 nM) with 250 μM Mn^2+^ at pH 8.0 was incubated with the indicated lysophospholipid substrate and the products detected by LC-MS/MS. *A*, the positional isomers present in the 16-LPG substrate. *B*, LPA formation by LpgDΔN hydrolysis of 16-LPG determined by LC-MS/MS. *C*, LPA formation by LpgDΔN hydrolysis of 1 to 16-LPG. *D*, LPA formation by LpgDΔN hydrolysis of 2 to 18:1-LPG. LpgDΔN (20 nM) with 250 μM Mn^2+^ at pH 8.0 was incubated with either LPG or cLPA and the reaction products detected by LC-MS/MS. *E*, cLPA formation by LpgDΔN hydrolysis of 16-LPG. *F*, LPA formation by LpgDΔN (20 nM) hydrolysis of 16-cLPA.

The 16-LPG (Avanti Polar Lipids) used as an LpgDΔN substrate separates into two peaks in LC-MS/MS workflow (Fig. 3*A*). The major peak is 1 to 16-LPG and the minor peak is the 2-acyl-LPG isomer. The acyl chains of lysophospholipids undergo spontaneous migration between the one- and 2-positions of the glycerol backbone, and at equilibrium they reach about 90% 1-position (24, 25, 26). The addition of LpgDΔN to this substrate led to a major reduction in the concentration of 1 to 16-LPG and the appearance of 1-LPA (Fig. 3*B*) suggesting that LpgDΔN was specific for 1-acyl-LPG. Acyl chain migration is base catalyzed (24, 25, 26), so isomerization is occurring in the LpgDΔN reaction at pH 8.0 accounting for the decrease in 2-acyl-LPG as it isomerizes to the 1-position and is degraded by LpgDΔN. The acyl chain positional selectivity of LpgDΔN was assessed in more detail by preparing pure 1-palmitoyl-LPG or 2-oleoly-LPG substrates in our laboratory from 1-palmitoyl-2-oleoyl-PG using bee venom phospholipase A2 or glycerol ester hydrolase, respectively (16). 1-Palmitoyl-LPG was converted to 1-palmitoyl-LPA by LpgDΔN (Fig. 3*C*), but LpgDΔN was inactive with 2-oleoyl-LPG as substrate (Fig. 3*D*). The small 1 to 18:1-LPG peak contaminant arising from isomerization of the 2 to 18:1-LPG was converted to LPA. These biochemical data explain why the inactivation of *lpgD* gene does not alter the levels of a15-LPG (Fig. 1), which exists as 2-a15-LPG in the cell (16).

#### Cyclic-lysophosphatidic acid (cLPA) formation

Both LPA and cLPA were products of LpgDΔN degradation of LPG (Fig. 3*E*). The LC-MS/MS standard curves showed that LPG and LPA were detected at the same efficiency, whereas cLPA detection was more efficient (Fig. S4*A*). Thus, cLPA was produced in lower abundance than LPA. cLPA was also a substrate for LpgDΔN (Fig. 3*F*). An LpgDΔN protein curve showed that LPA and cLPA are both formed at low protein concentrations and as the protein concentration increases the proportion of product that is cLPA decreases (Fig. S4*B*) because cLPA is also used as an LpgDΔN substrate. These data indicate that cLPA is an intermediate in the formation of LPA from LPG and leads to a two-step model for LpgDΔN catalysis (Fig. S4*C*). LPG binds to LpgD and cLPA is formed. Glycerol then dissociates from the enzyme allowing water to bind. cLPA is then hydrolyzed to LPA. cLPA can dissociate from the enzyme following glycerol release and can re-bind free LpgD and be converted to LPA (Fig. S4*C*).

#### Polar headgroup specificity

The only substrates available for LpgD in *S. aureus* are ≥16-LPG; however, the GlpQ family of phosphodiesterases is typically not specific for the type of headgroup attached to the glycerol backbone. LpgDΔN converted lysophosphatidylcholine (Fig. S5*A*), lysophosphatidylethanolamine (Fig. S5*B*), lysophosphatidylinositol (Fig. S5*C*), and lysophosphatidylserine (Fig. S5*D*) to a mixture of LPA and cLPA. Like other GlpQ family members, LpgD hydrolyzes a variety of lysophospholipids.

### Crystal structures of LpgDΔN

#### Overall protein fold

**Figure 4:**
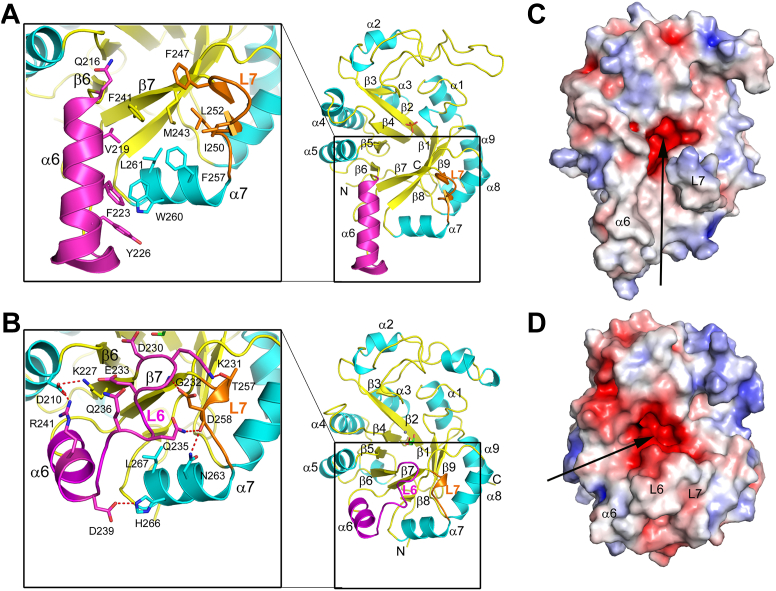
**X-ray crystal structure of LpgDΔN**. The structures were rendered with PyMOL (version 2.5.1, Schrödinger, LLC) with the helices in *cyan*, sheets, and loops in *yellow*, helix α6 in *magenta*, and loop L7 in *orange*. *A*, The 2.1 Å LpgDΔN structure (PDB ID: 8GHH). *Inset*, a close-up view of the area that differentiates LpgD from other GDPD structures. *B*, the 2.2 Å structure of SaGlpQ (PDB ID: 2OOG), a prototypical GDPD family member. *Inset*, a close-up view of the prototypical organization of helix α6 and loop L7 in the GDPD protein family. *C*, the electric potential surface of LpgDΔN illustrating the hydrophobic surface groove that leads to the active site created by helix α6 and loop L7 (*arrow*). *D*, the electric potential surface of SaGlpQ illustrating how helix α6, loop L6, and loop L7 interact to create a different, hydrophilic entrance to the active site (*arrow*). The electrostatic potential surface (from −5 to 5 kT/e) was rendered using the Adaptive Poisson-Boltzmann Solver (APBS) package within PyMOL. *Red* is negative, blue is positive, and *white* is neutral/hydrophobic potential.

His-tagged LpgDΔN produced crystals in space group I422 with a single molecule in the asymmetric unit in an acidic phosphate buffer (0.095 M phosphate-citrate, pH 5.3, 1.52 M sodium dihydrogen phosphate, and 0.38 M dipotassium hydrogen phosphate). The LpgDΔN structure was determined by molecular replacement using an AlphaFold structure as the search model and refined to 2.1 Å (Table S3; PDB ID: 8GHH). LpgDΔN has a β/α TIM barrel core structure composed of eight β-sheets surrounded by eight α-helices (27) that is found in an astonishing 10% of enzymes (28, 29) (Fig. 4*A*). LpgD belongs to the GDPD (GlpQ) subfamily of TIM barrels (Fig. S6). The root mean square deviation (RMSD) between the LpgDΔN structure and three GlpQ structures selected for comparison was ∼1 Å (Fig. S6) emphasizing the similarity of the LpgDΔN fold to other GDPD family members. The GDPD protein family is characterized by a TIM barrel modification that arises from the insertion of sequence elements between the second β-sheet and second α-helix of the symmetrical TIM barrel (30, 31). This feature is called the GDPD insert sequence (30, 31), and its length, sequence, and folding vary slightly between the known GDPD structures (Fig. S6). In all cases, the GDPD insert region makes an important contribution to catalysis by introducing and positioning a catalytically important histidine residue (His93 in LpgD) in the active site (30, 32).

#### A unique helix α6

The LpgDΔN structure is distinguished from the prototypical GDPD TIM barrel by the length, orientation, and hydrophobicity of helix α6 and loop L7 (Figs. 4 and S7). The 17-residue α6 of LpgDΔN is longer than the shorter α6 (8 residues) in SaGlpQ. The 11 remaining residues form the L6 loop of SaGlpQ pack against the protein surface making several polar sidechain interactions with the TIM barrel to fix the L6 conformation (Fig. 4*B*). The second feature in LpgDΔN that is different from SaGlpQ is loop L7 between β7 and α7 (Fig. S7). The L7 loop is longer in LpgDΔN (Fig. 4*A*) than in SaGlpQ (Fig. 4*B*). The net effect of the α6 and L7 structural elements in LpgDΔN is to create a hydrophobic surface grove that leads to the polar active site (Fig. 4*C*). In contrast, the interactions of L6 with the core TIM barrel and L7 in SaGlpQ fills the surface cavity present in LpgDΔN to create a hydrophilic path to the active site from a different direction (Fig. 4*D*). These distinguishing features reflect the differences in the structure of the substrates used by LpgD and SaGlpQ. SaGlpQ hydrolyzes hydrophilic glycerol phosphodiesters (33) and wall teichoic acid (glycerol phosphate) polymers (34, 35) as opposed to LpgD, which hydrolyzes glycerol phosphodiesters attached to long, hydrophobic acyl chain that must be accommodated by the protein.

#### Active site ligand

The 2Fo-Fc electron density map shows that there is a clearly defined dihydrogen phosphate (P1) located in the active site of LpgDΔN crystal plus a second phosphate (P2) connected to His93 (Fig. S8, *A* and *B*). P2 has a weaker electron density and a higher B factor than P1. The P1 phosphate in LpgDΔN is located in the same position in the active site as the phosphate of the glycerol phosphate ligand bound to *B. subtilis* GlpQ (Fig. S6*C*) suggesting that P1 is bound in LpgDΔN in the same manner as the substrate/product phosphate in GlpQ enzymes. We postulated that the actual ligand associated with His93 would most logically be a water molecule and that P2 was partially displacing the water due to the low pH and high phosphate concentration in the crystallization condition. Therefore, we obtained a second structure by soaking the LpgDΔN crystals with a pH 6.5 solution of 100 mM magnesium chloride, 0.1 M trisodium citrate, 1.0 M sodium dihydrogen phosphate, and 1.0 M dipotassium hydrogen phosphate (Table S3; PDB ID: 8GHI). The refined 2.4 Å structure showed a single dihydrogen phosphate ion (P1) located in the same position in the active site as was present in the initial, lower pH structure (Fig. S8*C*). However, the low occupancy phosphate (P2) is clearly replaced by a water molecule hydrogen bonded to His93 as illustrated by the Fo-Fc simulated annealing omit map (Fig. S8*D*). These data suggest His93 is hydrogen bonded to a water molecule in substrate-free LpgDΔN.

#### LPG binding

**Figure 5:**
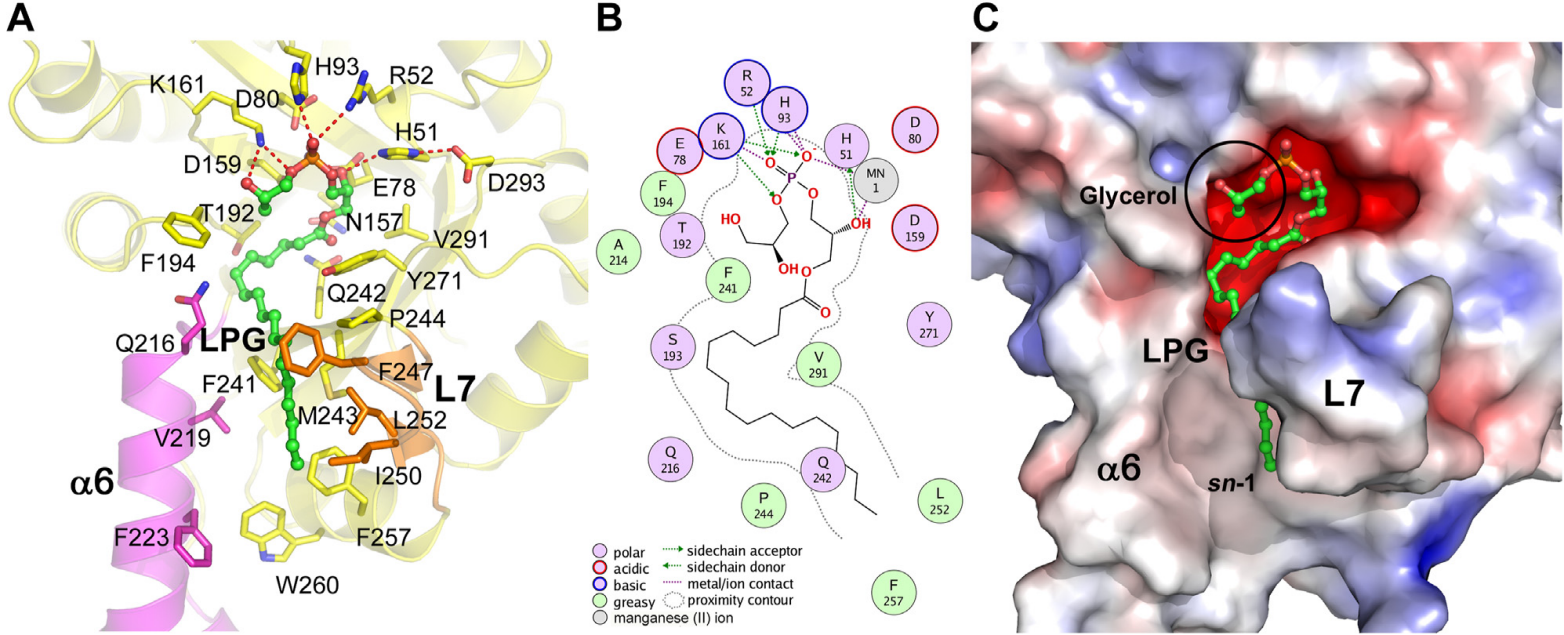
**Modeling substrate, intermediate, and products LpgD**. *A*, 1 to 18-LPG docked into the LpgDΔN active site illustrating the connections between substrate and active site residues. The acyl chain lies within the hydrophobic channel created by helix α6 (*magenta*) and loop L7 (*orange*). The molecular docking solution was calculated using the Molecular Operating Environment software (2018.01, Chemical Computing Group). The LpgDΔN structure (*yellow*) was rendered with PyMOL (version 2.5.1, Schrödinger, LLC) with helix α6 (*magenta*), loop L7 (*orange*), 1 to 18-LPG (*green*), and the hydrogen bonds depicted as *dashed red lines*. LpgDΔN residues predicted to interact with the LPG substrate are labeled. The 3′-hydroxyl of the LPG headgroup glycerol is obscured behind the glycerol *sn*-2 carbon in this image. *B*, two-dimensional protein-ligand interaction diagram for 1 to 18-LPG docked into LpgDΔN illustrating the connections between the substrate and active site residues. Ca^2+^ (*green ball*) sits behind the glycerol phosphate. *C*, surface view of 1 to 18-LPG docked into the LpgDΔN structure. The electrostatic potential surface (from −5 to 5 kT/e) was rendered using the Adaptive Poisson-Boltzmann Solver (APBS) package within PyMOL. *Red* is negative, blue is positive and *white* is neutral/hydrophobic potential. The glycerol headgroup of PG is *circled*.

**Figure 6:**
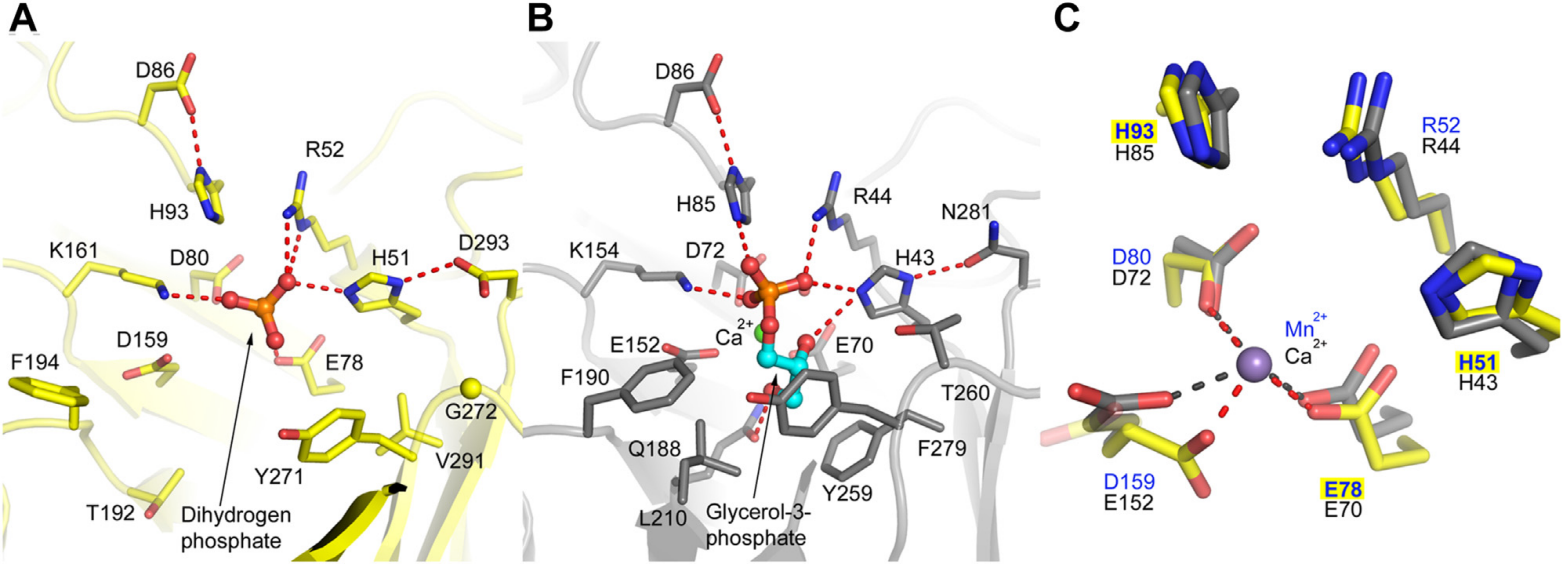
**Structure of the LpgDΔN active site**. *A*, a dihydrogen phosphate ion is found in the LpgDΔN active site (PDB ID: 8GHH). *B*, the active site of BsGlpQ (PDB ID: 5T9C) with the glycerol-3-phosphate product (*cyan*) and calcium ion (*green*). *C*, an overlay of the active sites of BsGlpQ (*grey*) and LpgDΔN (*yellow*) with the manganese ion modeled at the same location as calcium in BsGlpQ. The three active site residues mutated in LpgDΔN (Glu78, His93 and His51) are highlighted. The structures were rendered with PyMOL (version 2.5.1, Schrödinger, LLC) with the hydrogen bonds depicted as *dashed red lines*.

The molecular docking solution provided insight into how LpgD binds the LPG substrate (Fig. 5*A*). The polar glycerophosphoglycerol portion of LPG packs into a hydrophilic active site forming numerous hydrogen bond interactions with the side chains of residues within the active site (Fig. 5*B*). The LPG acyl chain extends out from the active site and lies along the hydrophobic surface groove created by helix α6 and loop-7 leading to the active site (Fig. 5*C*). The active site interactions of the P1 phosphate in LpgDΔN and the metal binding site were the same as observed in the BsGlpQ-glycerol phosphate complex structure (Fig. 6). The P1 dihydrogen phosphate in the LpgDΔN structure (Fig. 6*A*) forms the same hydrogen bond interactions with active site residues as are observed surrounding the phosphate in the glycerol-phosphate product structure of BsGlpQ (Fig. 6*B*). The LpgDΔN structure lacks a divalent cation; however, Mn^2+^ is proposed to bind to the same three acidic residues as the Ca^2+^ of BsGlpQ (Fig. 6*C*). Overall, the locations of the residues in the LpgDΔN active site are the same as found in other GlpQ family members suggesting a common mechanism for this group of enzymes.

### Site-directed mutagenesis

We prepared four LpgDΔN mutants in the active site residues highlighted in Figure 6*C*. All the mutant proteins were expressed and purified without any noticeable differences compared to wild-type LpgDΔN (Fig. S9*A*). All of the mutant proteins were as stable to thermal denaturation as LpgDΔN indicating that they were all properly folded proteins (Fig. S9*B*). These experiments were performed in the absence of Mn^2+^, and the LpgDΔN(E78A) mutant was actually more stable that wild-type (Fig. S9*B*). We also determined the shift in the thermal stabilities of the mutants caused by the addition of Mn^2+^ (Fig. S9*C*). All of the proteins were stabilized to thermal denaturation by Mn^2+^ except for LpgDΔN(E78A). These data show that the mutant proteins were properly folded monomers and confirms that the acidic residue cluster in the LpgDΔN active center as the Mn^2+^ binding site.

**Figure 7:**
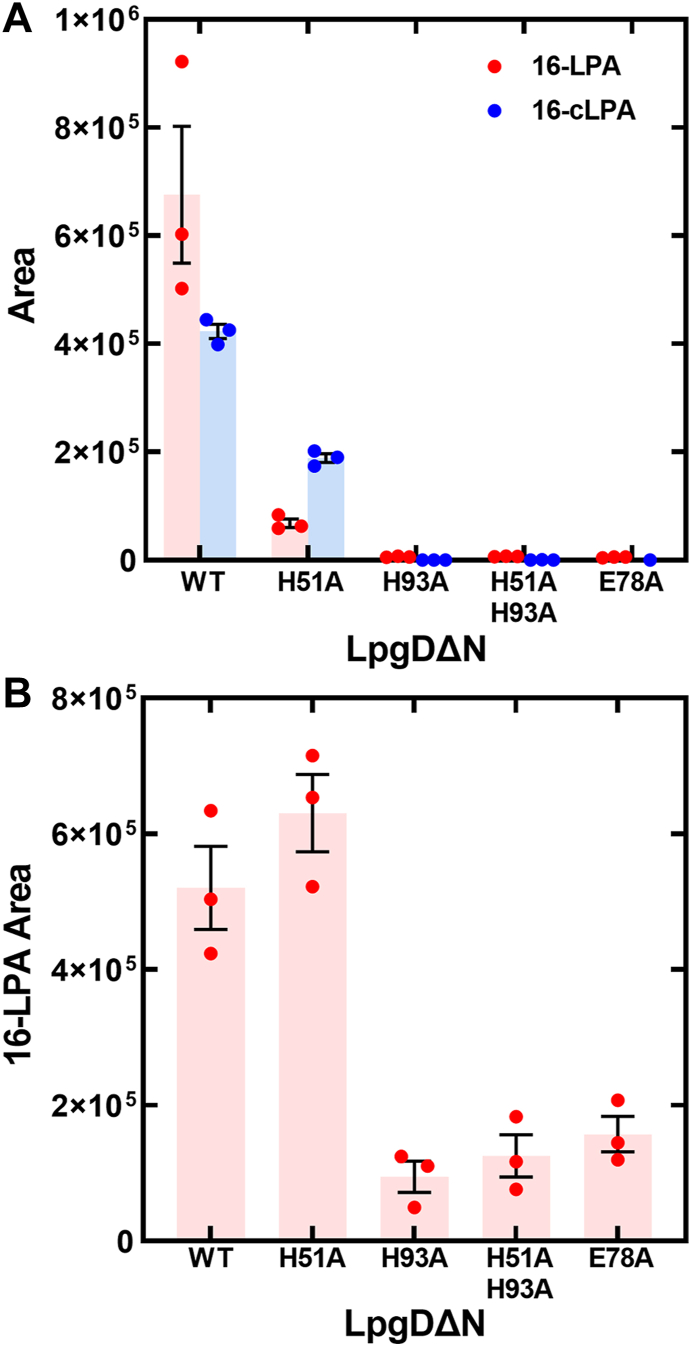
**Activities of LpgDΔN mutants using LPG and cLPA substrates.** LpgDΔN(H51A), LpgDΔN(E78A), LpgDΔN(H93A), and LpgDΔN(H51A,H93A) were purified (Fig. S9) and assayed for their ability to form 16-LPA and 16-cLPA from 16-LPG. *A*, activities of the LpgDΔN mutant panel using 16-LPG as substrate. *B*, activities of the LpgDΔN mutant panel using 16-cLPA as substrate.

The mutant LpgDΔN panel was tested for activity using LPG as substrate. The LpgDΔN(E78A), LpgDΔN(H93A), and LpgDΔN(H51A, H93A) mutants were deficient in the hydrolysis of LPG (Fig. 7*A*). Although LpgDΔN(H51A) activity was lower than LpgDΔN, LpgDΔN(H51A) retained significant catalytic activity indicating that His51 was a rate-accelerating residue but was not absolutely essential for catalysis (Fig. 7*A*). LpgDΔN(H51A) also had a higher proportion of cLPA product compared to LpgDΔN. Prior work with GlpQ showed that the residues corresponding to His93 and Glu78 in LpgD were also essential for catalysis; however, the role for His51 was not studied (31). LpgDΔN(H51A) was just as active in cLPA hydrolysis as LpgDΔN (Fig. 7*B*). LpgDΔN(E78A), LpgDΔN(H93A) and LpgDΔN(H51A, H93A) were compromised in the hydrolysis of cLPA to LPA but were nonetheless active with this substrate (Fig. 7*B*). These data show that His93 and the Mn^2+^ center are important for efficient catalysis of both LpgD half-reactions, whereas His51 is not needed to convert cLPA to LPA.

## Discussion

**Figure 8:**
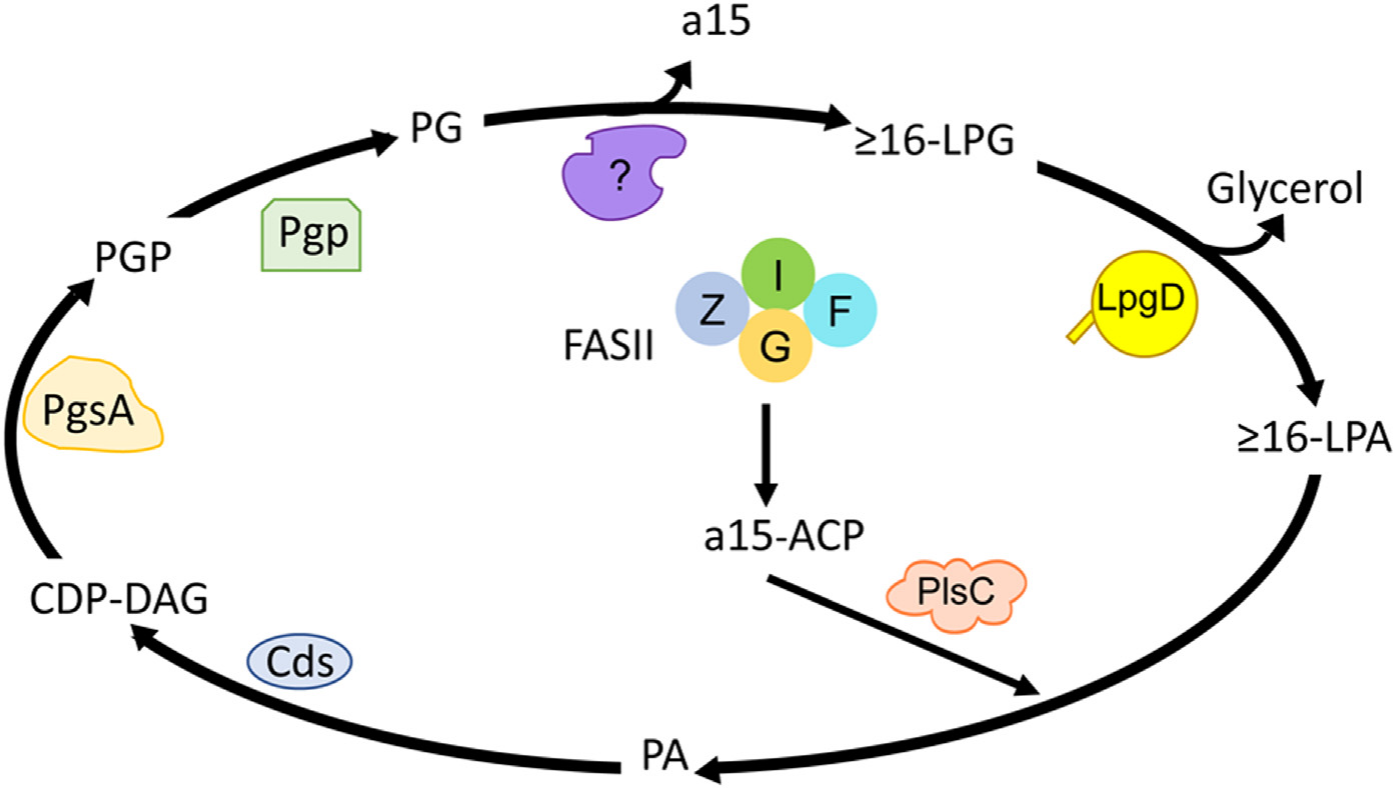
**The role of LpgD in *S. aureus* membrane phospholipid homeostasis.** The 1-acyl-LPG molecular species in *S. aureus* have ≥16-carbon acyl chains (≥16-LPG) and arise from unknown metabolic process(es) that remove a15 from the 2-position of PG (?). LpgD converts ≥16-LPG to ≥16-LPA. LPA is the substrate for LPA acyltransferase (PlsC), which is highly specific for *anteiso*-pentadecanoyl-ACP (a15-ACP) arising from the fatty acid biosynthetic pathway (FASII). The phosphatidic acid (PA) is converted in three enzymatic steps to PG. (Cds, CDP-diacylglycerol synthase; PgsA, PG phosphate synthase; and Pgp, PG phosphate phosphatase). The LpgD cycle resynthesizes the same molecular species of bilayer-forming PG that were degraded by the processes that created ≥16-LPG.

This study identifies LpgD as a lysophospholipase D that catalyzes the conversion of 1-acyl-LPG generated in *S. aureus* to LPA which is re-introduced into the PG biosynthetic pathway at the PlsC step (Fig. 8). *S. aureus* phospholipids consist of ≥16-carbon acyl chains in the 1-position paired with a15 fatty acid in the 2-position (14, 15). Although the enzyme or biological process that creates 1-(≥16)-LPG in *S. aureus* remains unknown, the large impact of the *lpgD* deletion mutant on the intra- and extracellular concentrations of ≥16-LPG molecular species speaks to the importance of LpgD in maintaining low concentrations of membrane 1-acyl-lysophospohlipids. The conversion of ≥16-LPG into ≥16-LPA by LpgD is the first step in recycling the lysophospholipids into the membrane phospholipid biosynthetic pathway (Fig. 8). The LPA is then acylated by PlsC to re-introduce a15 into the 2-position and the ≥16/a15-phosphatidic acid is converted to PG in three steps (Fig. 8). In mammals deacylation–reacylation cycles involving lysophospholipid intermediates are responsible for the remodeling of the membrane phospholipid molecular species composition (4). However, in *S. aureus*, the LpgD cycle is not used to remodel the membrane but rather regenerates the same mixture of PG molecular species that was present when the ≥16-LPG was produced (Fig. 8).

**Figure 9:**
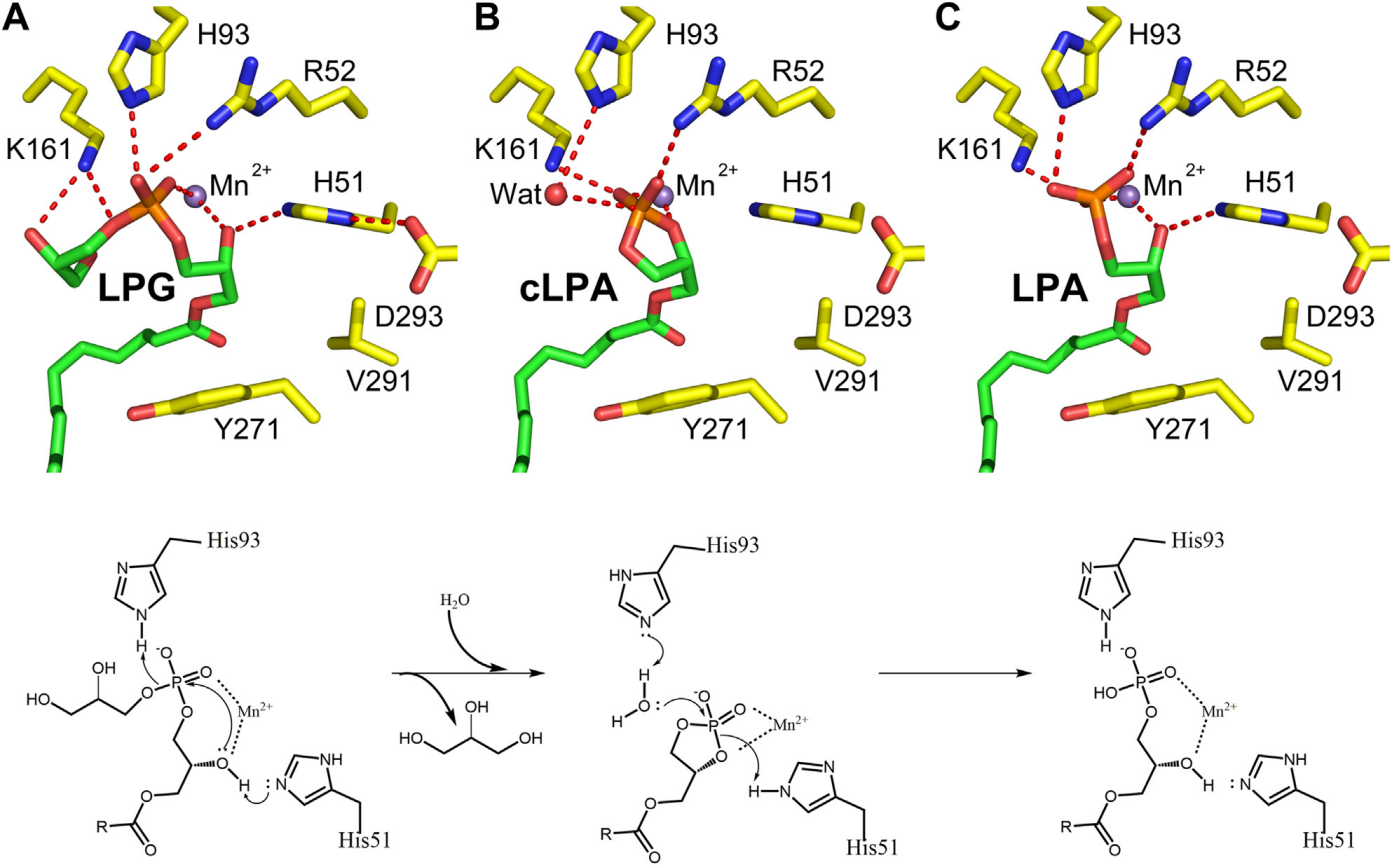
**The role of active site residues in the formation and hydrolysis of the cLPA LpgD reaction intermediate**. *A*, the LPG substrate docked into the LpgD active site illustrating its interactions with sidechains and Mn^2+^. His51 and Mn^2+^ activate the *sn*-2-hydroxyl of LPG to attack the phosphodiester and His93 donates a proton to facilitate the release of the glycerol headgroup. Glycerol dissociates from LpgD and water enters the active site. *B*, the cLPA intermediate is bound by the same LpgD sidechains and Mn^2+^. His93 activates the incoming water molecule to attack the cyclic phosphate of cLPA. His51, no direct interaction with cLPA, donates a proton to the *sn*-2 hydroxyl to facilitate LPA formation. *C*, the product complex shows His51 reengages the *sn*-2 hydroxyl LPA. Molecular docking solutions were calculated using the Molecular Operating Environment software (2018.01, Chemical Computing Group). The LpgDΔN structure (*yellow*) with the bound 1 to 18-LPG (*green*) was rendered with PyMOL (version 2.5.1, Schrödinger, LLC) and the hydrogen bonds were depicted as *dashed red lines*. The chemical reactions were drawn with ChemDraw Professional (version 17.1, PerkinElmer Informatics).

This study establishes that LpgD acts by a two-step mechanism employing a cLPA intermediate (Fig. 9). Several investigators have suggested that TIM barrel phosphodiesterases have a two-step mechanism. One proposal was that the first product formed is a cyclic-phosphate intermediate that is subsequently hydrolyzed by the addition of water (30, 36, 37). Others suggest that the reaction proceeds *via* a phosphohistidine intermediate (37, 38) analogous to the phosphohistidine intermediate formed in the phospholipase D family of enzymes that form phosphatidic acid from phospholipids (39, 40). Neither of these intermediates has been demonstrated in the GlpQ-like enzymes; however, toxins from scicariid spiders are TIM barrel phospholipase Ds that are distantly related to the GDPD enzyme family (37, 38, 41, 42). These proteins have an LpgD/GlpQ-like dual histidine/metal ion active sites, but only produce cyclicphosphoceramide from sphingomyelin, and to a lesser extent, cLPA from LPC (43, 44). Our demonstration that cLPA is both formed and degraded by LpgD establishes the mechanism of the GDPD enzyme group. LPG binds to the free enzyme with His51 and the Mn^2+^ cluster activating the 2-hydroxyl of LPG and His93 positioned to act as a general acid to facilitate the release of glycerol and the formation of the cLPA intermediate (Fig. 9*A*). Molecular modeling suggests that there is no room in the active site for both water and the glycerol headgroup of LPG to simultaneously bind LpgD (Fig. 9*A*). The release of glycerol allows water to enter the active site and His93 acts as general base that catalyzes the water addition to the cLPA intermediate (Fig. 9*B*) to form the LpgD-LPA product complex (Fig. 9*C*). The P1 phosphate in the LpgDΔN structure is located in a similar position as the phosphate of the substrate LPG, cLPA intermediate and LPA product complex docking solutions (Fig. 9) and in the *B. subtilis* GlpQ structure (Fig. S6*C*). His93 is the key histidine for both steps in the reaction as supported by the compromised hydrolysis of both LPG and cLPA by the LpgDΔN(H93A) mutant. The LpgDΔN(H51A) mutant is defective in LPA/cLPA formation from LPG but has wild-type cLPA hydrolysis activity supporting the model (Figs. 7*B* and 9*B*). Based on the strong structural similarities between LpgD and other GDPD family members, we conclude that a cyclic-phosphate intermediate is common to the GDPD group of phosphodiesterases (Fig. 9).

Our analysis of LpgD also advances the understanding of two human GDPD family proteins, GDE4 (GDPD1) and GDE7 (GDPD3). These proteins are known as lysophospholipase D enzymes whose biological roles are under active investigation. GDE4 interacts with heterotrimeric G protein subunits (45, 46), and both GDE4 and GDE7 generate N-acylethanolamine from N-acyl-lysophosphatidylethanolamines (47, 48). Regulation of LPA levels by GDE7 has a role in liver lipid homeostasis (49) and the maintenance of leukemia stem cells (50). GDE4 binds Mn^2+^ and is most active in hydrolyzing LPG to LPA, whereas GDE7 binds Ca^2+^ and hydrolyzes LPC to a mixture of LPA and cLPA (51, 52). The structural similarities between LpgD and the two human proteins are striking. All three proteins have an amino-terminal α-helix that is proposed to anchor the enzymes to the membrane. The idea that GDE4 has two transmembrane regions based on primary sequence analysis (53) is not consistent with our LpgDΔN structure or the AlphaFold predictions (Fig. S10). The RMSD between LpgD and its AlphaFold structure is 0.794 Å (Fig. S10*A*). The only slight difference in the two structures is in the orientation of helix α6, which has a slightly more open conformation in LpgDΔN compared to its AlphaFold prediction (Fig. S10*A*). The RMSDs between the LpgDΔN structure and the AlphaFold structures of GDE4 (Fig. S10*B*) and GDE7 (Fig. S10*C*) are 1.007 Å and 0.873 Å, respectively. This high degree of overall structural similarity and the same constellation of active site residues in all enzymes suggests that these proteins have the same catalytic mechanism as LpgD.

## Experimental procedures

### Materials and strains

All chemicals and reagents were reagent grade or better. Heavy [d5]17-LPG was purchased from Cambridge Isotope Laboratories, Inc. 14-LPG, 16-LPG, 16-LPC, 16-LPE, 16-LPS, 16-LPI, 16-cLPA, and 1-palmitoyl-2-oleoyl-*sn*-glycero-3-phospho-(1′-*rac*-glycerol) (Avanti Polar Lipids), Sypro Orange dye (Invitrogen). *S. aureus* strains used in the study are listed in Table S1. *S. aureus* strains were routinely grown in Luria broth (10 g tryptone, 5 g yeast extract, 5 g NaCl per liter).

### Molecular biology

For the generation of Δ*lpgD* in AH1263 (PDJ73), deletion plasmids were constructed by cloning the areas upstream and downstream of the region to be deleted into the temperature-sensitive *E. coli*-*S. aureus* shuttle vector pJB38 and allelic exchange were performed as described in (54). The *lpgD* gene minus the amino-terminal helix (Δ1-24) was amplified by PCR using primers designed for Gibson Assembly cloning into NcoI and XhoI digested pET28a to express LpgDΔN with a carboxy-terminal His-tag. The full length *lpgD* and *lpgD*Δ*N* genes with engineered carboxy-terminal His-tags were cloned into the CdCl_2_-inducible plasmid pCN51 by Gibson Assembly into PstI and EcoRI digested pCN51 to get pPJ659 and pPJ660, respectively. Plasmids were rendered transformable into normal *S. aureus* strains by passing them through strain RN4220. These plasmids were transformed into AH1263 and PDJ73 for complementation. LpgDΔN(E78A), LpgDΔN(H51A), and LpgDΔN(H93A) were generated by site-directed mutagenesis using the Quick-change Lightning kit (Agilent) in pP654 to obtain pPJ663, pPJ661, and pPJ664, respectively. The LpgDΔN(H51A,H93A) double mutant in pPJ662 was constructed from plasmid pPJ654 using Quick-change Lightning Multi kit (Agilent) on pPJ654. A list of plasmids used in the study are given in Table S1.

### LPG extraction

All strains listed in Table S1 were grown in LB to an A_600_ of 4.0. LPG was extracted from 5 ml of cells or 1 ml of 0.22 μm filtered media supernatant. The cells were resuspended in 0.5 ml water and 0.5 ml of cold methanol containing 1% acetic acid was added. To the 1 ml of filtered media, 1 ml of cold methanol containing 1% acetic acid was added. Samples were incubated on ice for 10 min and centrifuged at 20,000g for 20 min. Supernatants were dried in a speed vac and resuspended in 80% methanol containing 100 ng/ml of [d5]17-LPG for quantification. LPG was extracted from cells and media as described above following the growth of the culture in LB to an OD_600_ of 0.3 and induced with 6 μM CdCl_2_ for 4 h.

### Mass spectrometry

The 1-acyl- and 2-acyl-LPGs are separated from each other in the liquid chromatography fractionation step in the LC-MS/MS workflow with the 2-acyl isomer eluting first in the gradient (55). We used a similar system to validate the elution positions of the isomers and the LPG MRM transitions are listed therein (16). Briefly, LPG was analyzed using a Shimadzu Prominence UFLC attached to a QTrap 4500 equipped with a Turbo V ion source (Sciex). Samples were injected onto an Acquity UPLC HSS C18, 2.5 μm, 3.0 × 150 mm column at 30 °C (Waters) using a flow rate of 0.2 ml/min. Solvent A was 5 mM ammonium acetate + 0.1% formic acid, and Solvent B was 95% methanol + 5 mM ammonium acetate + 0.1% formic acid. The HPLC program was the following: starting solvent mixture of 35% A/65% B, 0 to 1 min isocratic with 65% B; 1 to 3 min linear gradient to 100% B; 3 to 30 min isocratic with 100% B; 30 to 32 min linear gradient to 65% B; 32 to 35 min isocratic with 65% B. The QTrap 4500 was operated in the negative mode, and the ion source parameters were: ion spray voltage, −4500 V; curtain gas, 30 psi; temperature, 500 °C; collision gas, medium; ion source gas 1, 20 psi; ion source gas 2, 35 psi; decluttering potential, −80 V; and collision energy, −30 V. [d5]17-LPG was used as the internal standard. The system was controlled by the Analyst software (Sciex) and analyzed with MultiQuant 3.0.2 software (Sciex). Peaks corresponding to individual LPG species were quantified relative to the internal standard.

### Purification of LpgDΔN

pPJ654, pPJ663, pPJ661, pPJ664, and pPJ662, were transformed into BL21(DE3) cells and grown in LB to an OD_600_ of 0.7 at 37 °C with shaking at 200 rpm. The culture was cooled to 16 °C and the cells were induced with 1 mM IPTG overnight with shaking at 200 rpm. Cells were harvested and resuspended in 20 mM Tris (pH 8.0), 200 mM NaCl, and 10 mM imidazole. Cells were broken by two passages through a cell disruptor and centrifuged at 20,000*g* for 45 min. LpgDΔN was purified from the supernatant using a Ni-NTA column by washing with 20 column volumes of each 20 mM Tris (pH 8.0), 200 mM NaCl containing 10 mM imidazole or 20 mM imidazole or 40 mM Imidazole. LpgDΔN was eluted with 20 mM Tris (pH 8.0), 200 mM NaCl, 250 mM imidazole. The eluant was dialyzed with 20 mM Tris (pH 7.5), 500 mM NaCl and 100 mM EDTA. This was further dialyzed with 20 mM Tris (pH 7.5), 400 mM NaCl and 50 mM EDTA for 4 h, then with 20 mM Tris (pH 7.5), 300 mM NaCl and 25 mM EDTA for 4 h and finally with 20 mM Tris (pH 7.5), 200 mM NaCl and no EDTA overnight. The molecular weight was calculated by analyzing the elution position on a 10/300 Gl Superdex 200 analytical column (Cytiva Life Sciences) eluted with 20 mM Tris, (pH 7.5), 200 mM NaCl. A standard curve was generated using thyroglobulin (669 kDa), ferritin (440 kDa), aldolase (158 kDa), conalbumin (75 kDa), ovalbumin (44 kDa), ribonuclease A (13.7 kDa), and aprotinin (6.5 kDa).

### LpgD assays

The *in vitro* activity of LpgD was analyzed by mass spectrometry. The assay mixture containing 100 mM Tris (pH 8.0), 150 mM NaCl, 250 μM MnCl_2_, 10 μM 14-LPG was treated with LpgD(ΔN) at 20 nM for 20 min at 37 °C. The reaction was stopped by adding 50 μl of methanol containing 1% acetic acid. The rection was centrifuged at 20,000*g* for 20 min, and the samples were analyzed by LC-MS/MS. For assays with other divalent cations 1 mM MnCl_2_ was replaced with 1 mM MgCl_2_ or CaCl_2_ or ZnCl_2_ at concentration as indicated in the figure. To test different substrates 14-LPG was replaced with 16-LPG, 16-LPC, 16-LPE, 16-LPS, 16-LPI, or 16-cLPA at the concentration indicated in the figure. To obtain 1 to 16:0-LPG or 2 to 18:1-LPG, liposomes made with 1-palmitoyl-2-oleoyl-*sn*-glycero-3-phospho-(1′-*rac*-glycerol) were treated with 0.1 μg/ml of PLA2 from bee venom or 50 nM Geh (16), respectively, in a reaction containing 1 μM LpgDΔN for 1 h. Samples were processed and analyzed as described earlier.

### Thermal stability measurements

Purified LpgDΔN and mutants LpgDΔN(E78A), LpgDΔN(H51A), LpgDΔN(H93A) and LpgDΔN(H51A,H93A) were subjected to thermal denaturation in the presence of Sypro Orange dye as described in (56). Briefly, 10 μM of each of the proteins were added to 30 μl of 100 mM HEPES (pH 7.5) and 5X Sypro Orange dye in a 96-well plate. The stability of the proteins in the presence of divalent cations was done in the presence of 100 μM EDTA plus 10 mM MnCl_2_ or MgCl_2_. The plate was centrifuged at 1500 rpm for 2 min before being placed in an Applied Biosciences 7500 RealTime PCR instrument. A thermal scan from 25 °C to 95 °C was performed using an increment rate of 1 °C/min. All experiments were performed in triplicate.

### Sedimentation velocity

Sedimentation velocity experiments were conducted in a ProteomeLab XL-I analytical ultracentrifuge (Beckman Coulter) with an AnTi-50 eight-hole rotor following standard protocols unless mentioned otherwise (57). Samples in buffer containing 20 mM Tris, pH 7.0, 200 mM NaCl 0.5 mM EDTA 5 mM BME were loaded into cell assemblies with double sector 12 mm centerpieces and sapphire windows. The cell assemblies, containing identical sample and reference buffer volumes of 320 μl, were placed in a rotor. After temperature equilibration at nominal 20 °C, the rotor was accelerated to 50,000 rpm, and Rayleigh interference optical data were collected. The velocity data were analyzed with the sedimentation coefficient distribution model *c(s)* in SEDFIT (58). The signal-average frictional ratio and meniscus position were refined with non-linear regression and maximum entropy regularization was applied at a confidence level of *p* = 0.68. Buffer densities and viscosities at 20 °C were measured using a densitometer model DMA 5000 M and a micro-viscometer model AMVn respectively (both from Anton Paar Inc., Ashland, VA), and calculated using the software SEDNTERP (59).

### X-ray crystallography

LpgDΔN protein crystals (PDB ID: 8GHH) were grown by the sitting drop vapor diffusion method at 20 °C with a reservoir solution of 0.095 M phosphate-citrate, pH 5.3, 1.52 M sodium dihydrogen phosphate, and 0.38 M di-potassium hydrogen phosphate. The sitting drops were produced by mixing 0.2 μl of protein solution (17.5 mg/ml in 20 mM Tris-HCl, pH 6.5, 0.2 M NaCl, and 10 mM MgCl2) with 0.2 μl of reservoir solution using a Mosquito crystallization robot (TTP Labtech). Crystals were transferred into a cryo-protection solution (0.1 M phosphate-citrate, pH 5.3, 1.6 M sodium dihydrogen phosphate, 0.4 M di-potassium hydrogen phosphate, 5 mM MgCl_2_, and 30 % glycerol) and flash frozen in liquid nitrogen. Diffraction data were collected at the SERCAT beam line 22-BM at the Advanced Photon Source and processed using HKL-2000 (60) and analyzed using Xtriage (61). The LpgDΔN structure was solved by molecular replacement method using Phaser Crystallographic Software (62). The search model was produced by AlphaFold using ChimeraX and ColabFold (63, 64, 65). A second structure was obtained (PDB ID: 8GHI) by soaking the LpgDΔN crystals with a pH 6.5 solution (100 mM MgCl_2_, 0.1 M trisodium citrate, 1.0 M sodium dihydrogen phosphate, and 1.0 M di-potassium hydrogen phosphate) for 2 days. These crystals were transferred into a cryo-protection solution (0.1 M trisodium citrate, 1.0 M sodium dihydrogen phosphate, 1.0 M di-potassium hydrogen phosphate, 100 mM MgCl_2_, and 32% glycerol) and flash frozen in liquid nitrogen. Diffraction data were collected at the SERCAT beam line 22-ID at the Advanced Photon Source. The structures were refined and optimized using PHENIX (66) and COOT (67), respectively. Data collection and refinement statistics are summarized in the Table S3. Figures were rendered with PyMOL (version 2.5.1, Schrödinger, LLC).

### Molecular docking

Molecular docking of 1 to 18-LPG substrate with LpgDΔN protein was performed using Molecular Operating Environment (MOE) software (2018.01; Chemical Computing Group). For docking calculation, the LPG ligand was drawn using ChemDraw Professional and converted to a 3D model (version 17.1, PerkinElmer Informatics). Energy minimization was performed with Chem3D Ultra (version 17.1, PerkinElmer Informatics) using the force fields MMFF94. The manganese ion-modeled LpgDΔN structure was generated using the BsGlpQ complex structure (PDB ID: 5T9C). After superimposing the LpgDΔN and BsGlpQ structures using MachMaker in ChimeraX, the manganese ion in LpgDΔN was placed at the calcium ion site of BsGlpQ. Asp159 residue was switched to another rotamer to interact with the manganese ion. The ligand and protein structures were imported into MOE, and partial charges were added to the ligand structure. The protein structure was prepared using the “QuickPrep” function and NE2 atom of His93 was protonated. After docking was performed using the “Dock” function in MOE, a docking model with the top conformation was identified based on S-score binding affinity. The cLPA and LPA structures were generated from the docking model of 18-LPG using Builder Mode in MOE. The cLPA and LPA structures were subjected to energy minimization using MOE with the NE2 atoms of both His 51 and His93 in their protonated forms.

## Data availability

The LpgDΔN structural coordinates are deposited in the Protein Data Bank (accession numbers 8GHI and 8GHH). All other data produced for this work are contained within the article.

## Supporting information

This article contains supporting information (14, 18, 19).

## Conflict of interest

The authors declare that they have no conflicts of interest with the contents of this article.

## References

[1] Zhang Y.-M., Rock C.O. (2008). Membrane lipid homeostasis in bacteria. Nat. Rev. Microbiol..

[2] Tan S.T., Ramesh T., Toh X.R., Nguyen L.N. (2020). Emerging roles of lysophospholipids in health and disease. Prog. Lipid Res..

[3] Kano K., Aoki J., Hla T. (2022). Lysophospholipid mediators in health and disease. Annu. Rev. Pathol..

[4] Valentine W.J., Yanagida K., Kawana H., Kono N., Noda N.N., Aoki J. (2022). Update and nomenclature proposal for mammalian lysophospholipid acyltransferases, which create membrane phospholipid diversity. J. Biol. Chem..

[5] Zheng L., Lin Y., Lu S., Zhang J., Bogdanov M. (2017). Biogenesis, transport and remodeling of lysophospholipids in Gram-negative bacteria. Biochim. Biophys. Acta Mol. Cell Biol. Lipids.

[6] Snijder H.J., Ubarretxena-Belandia I., Blaauw M., Kalk K.H., Verheij H.M., Egmond M.R. (1999). Structural evidence for dimerization-regulated activation of an integral membrane phospholipase. Nature.

[7] Kaplan-Harris L., Weiss J., Mooney C., Beckerdite-Quagliata S., Elsbach P. (1980). The action of human and rabbit serum phospholipase A2 on *Escherichia coli* phospholipids. J. Lipid Res..

[8] Gupta S.D., Dowhan W., Wu H.C. (1991). Phosphatidylethanolamine is not essential for the N-acylation of apolipoprotein in *Escherichia coli*. J. Biol. Chem..

[9] Buddelmeijer N., Young R. (2010). The essential *Escherichia coli* apolipoprotein N-acyltransferase (Lnt) exists as an extracytoplasmic thioester acyl-enzyme intermediate. Biochemistry.

[10] Harvat E.M., Zhang Y.-M., Tran C.V., Zhang Z., Frank M.W., Rock C.O. (2005). Lysophospholipid flipping across the *Escherichia coli* inner membrane catalyzed by a transporter (LplT) belonging to the major facilitator superfamily. J. Biol. Chem..

[11] Cooper C.L., Hsu L., Jackowski S., Rock C.O. (1989). 2-Acylglycerolphosphoethanolamine acyltransferase/acyl-acyl carrier protein synthetase is a membrane-associated acyl carrier protein binding protein. J. Biol. Chem..

[12] Hsu L., Jackowski S., Rock C.O. (1991). Isolation and characterization of *Escherichia coli* K-12 mutants lacking both 2-acyl-glycerophosphoethanolamine acyltransferase and acyl-acyl carrier protein synthetase activity. J. Biol. Chem..

[13] Gardiner J.H.t., Komazin G., Matsuo M., Cole K., Gotz F., Meredith T.C. (2020). Lipoprotein N-acylation in *Staphylococcus aureus* is catalyzed by a two-component acyl transferase system. mBio.

[14] Parsons J.B., Frank M.W., Subramanian C., Saenkham P., Rock C.O. (2011). Metabolic basis for the differential susceptibility of Gram-positive pathogens to fatty acid synthesis inhibitors. Proc. Natl. Acad. Sci. U. S. A..

[15] Frank M.W., Whaley S.G., Rock C.O. (2021). Branched-chain amino acid metabolism controls membrane phospholipid structure in *Staphylococcus aureus*. J. Biol. Chem..

[16] Subramanian C., Rock C.O. (2023). The phospholipase A1 activity of Glycerol Ester Hydrolase (Geh) is responsible for extracellular 2-12(S)-methyltetradecanoyl-lysophosphatidylglycerol Production in Staphylococcus aureus. mSPhere.

[17] Ericson M.E., Subramanian C., Frank M.W., Rock C.O. (2017). Role of fatty acid kinase in cellular lipid homeostasis and SaeRS-dependent virulence factor expression in *Staphylococcus aureus*. mBio.

[18] Fey P.D., Endres J.L., Yajjala V.K., Widhelm T.J., Boissy R.J., Bose J.L. (2013). A genetic resource for rapid and comprehensive phenotype screening of nonessential *Staphylococcus aureus* genes. mBio.

[19] Charpentier E., Anton A.I., Barry P., Alfonso B., Fang Y., Novick R.P. (2004). Novel cassette-based shuttle vector system for gram-positive bacteria. Appl. Environ. Microbiol..

[20] Teufel F., Almagro Armenteros J.J., Johansen A.R., Gislason M.H., Pihl S.I., Tsirigos K.D. (2022). SignalP 6.0 predicts all five types of signal peptides using protein language models. Nat. Biotechnol..

[21] Yanaka N. (2007). Mammalian glycerophosphodiester phosphodiesterases. Biosci. Biotechnol. Biochem..

[22] Corda D., Mosca M.G., Ohshima N., Grauso L., Yanaka N., Mariggio S. (2014). The emerging physiological roles of the glycerophosphodiesterase family. FEBS J..

[23] Larson T.J., Ehrmann M., Boos W. (1983). Periplasmic glycerophosphodiester phosphodiesterase of *Escherichia coli*, a new enzyme of the *glp* regulon. J. Biol. Chem..

[24] Pluckthun A., Dennis E.A. (1982). Acyl and phosphoryl migration in lysophospholipids: importance in phospholipid synthesis and phospholipase specificity. Biochemistry.

[25] Kielbowicz G., Smuga D., Gladkowski W., Chojnacka A., Wawrzenczyk C. (2012). An LC method for the analysis of phosphatidylcholine hydrolysis products and its application to the monitoring of the acyl migration process. Talanta.

[26] Sugasini D., Subbaiah P.V. (2017). Rate of acyl migration in lysophosphatidylcholine (LPC) is dependent upon the nature of the acyl group. Greater stability of sn-2 docosahexaenoyl LPC compared to the more saturated LPC species. PLoS One.

[27] Romero-Romero S., Kordes S., Michel F., Hocker B. (2021). Evolution, folding, and design of TIM barrels and related proteins. Curr. Opin. Struct. Biol..

[28] Copley R.R., Bork P. (2000). Homology among (beta, alpha)(8) barrels: Implications for the evolution of metabolic pathways. J. Mol. Biol..

[29] Nagano N., Orengo C.A., Thornton J.M. (2002). One fold with many functions: the evolutionary relationships between TIM barrel families based on their sequences, structures and functions. J. Mol. Biol..

[30] Santelli E., Schwarzenbacher R., McMullan D., Biorac T., Brinen L.S., Canaves J.M. (2004). Crystal structure of a glycerophosphodiester phosphodiesterase (GDPD) from Thermotoga maritima (TM1621) at 1.60 A resolution. Proteins.

[31] Shi L., Liu J.F., An X.M., Liang D.C. (2008). Crystal structure of glycerophosphodiester phosphodiesterase (GDPD) from *Thermoanaerobacter tengcongensis*, a metal ion-dependent enzyme: insight into the catalytic mechanism. Proteins.

[32] Myers C.L., Li F.K., Koo B.M., El-Halfawy O.M., French S., Gross C.A. (2016). Identification of two phosphate starvation-induced wall teichoic acid hydrolases provides first insights into the degradative pathway of a key bacterial cell wall component. J. Biol. Chem..

[33] Jorge A.M., Schneider J., Unsleber S., Gohring N., Mayer C., Peschel A. (2017). Utilization of glycerophosphodiesters by *Staphylococcus aureus*. Mol. Microbiol..

[34] Jorge A.M., Schneider J., Unsleber S., Xia G., Mayer C., Peschel A. (2018). *Staphylococcus aureus* counters phosphate limitation by scavenging wall teichoic acids from other staphylococci *via* the teichoicase GlpQ. J. Biol. Chem..

[35] Walter A., Unsleber S., Rismondo J., Jorge A.M., Peschel A., Grundling A. (2020). Phosphoglycerol-type wall and lipoteichoic acids are enantiomeric polymers differentiated by the stereospecific glycerophosphodiesterase GlpQ. J. Biol. Chem..

[36] Friedman P., Haimovitz R., Markman O., Roberts M.F., Shinitzky M. (1996). Conversion of lysophospholipids to cyclic lysophosphatidic acid by phospholipase D. J. Biol. Chem..

[37] Murakami M.T., Fernandes-Pedrosa M.F., Tambourgi D.V., Arni R.K. (2005). Structural basis for metal ion coordination and the catalytic mechanism of sphingomyelinases D. J. Biol. Chem..

[38] Murakami M.T., Fernandes-Pedrosa M.F., de Andrade S.A., Gabdoulkhakov A., Betzel C., Tambourgi D.V. (2006). Structural insights into the catalytic mechanism of sphingomyelinases D and evolutionary relationship to glycerophosphodiester phosphodiesterases. Biochem. Biophys. Res. Commun..

[39] Gottlin E.B., Rudolph A.E., Zhao Y., Matthews H.R., Dixon J.E. (1998). Catalytic mechanism of the phospholipase D superfamily proceeds *via* a covalent phosphohistidine intermediate. Proc. Natl. Acad. Sci. U. S. A..

[40] Leiros I., McSweeney S., Hough E. (2004). The reaction mechanism of phospholipase D from Streptomyces sp. strain PMF. Snapshots along the reaction pathway reveal a pentacoordinate reaction intermediate and an unexpected final product. J. Mol. Biol..

[41] Lajoie D.M., Roberts S.A., Zobel-Thropp P.A., Delahaye J.L., Bandarian V., Binford G.J. (2015). Variable substrate preference among phospholipase D toxins from sicariid spiders. J. Biol. Chem..

[42] de Giuseppe P.O., Ullah A., Silva D.T., Gremski L.H., Wille A.C., Chaves Moreira D. (2011). Structure of a novel class II phospholipase D: Catalytic cleft is modified by a disulphide bridge. Biochem. Biophys. Res. Commun..

[43] Lajoie D.M., Cordes M.H. (2015). Spider, bacterial and fungal phospholipase D toxins make cyclic phosphate products. Toxicon.

[44] Lajoie D.M., Zobel-Thropp P.A., Kumirov V.K., Bandarian V., Binford G.J., Cordes M.H. (2013). Phospholipase D toxins of brown spider venom convert lysophosphatidylcholine and sphingomyelin to cyclic phosphates. PLoS One.

[45] Aoyama C., Sugimoto H., Ando H., Yamashita S., Horibata Y., Sugimoto S. (2011). The heterotrimeric G protein subunits Gα(q) and Gβ(1) have lysophospholipase D activity. Biochem. J..

[46] Aoyama C., Horibata Y., Ando H., Mitsuhashi S., Arai M., Sugimoto H. (2019). Characterization of glycerophosphodiesterase 4-interacting molecules Gαq/11 and Gβ, which mediate cellular lysophospholipase D activity. Biochem. J..

[47] Tsuboi K., Okamoto Y., Rahman I.A., Uyama T., Inoue T., Tokumura A. (2015). Glycerophosphodiesterase GDE4 as a novel lysophospholipase D: a possible involvement in bioactive N-acylethanolamine biosynthesis. Biochim. Biophys. Acta.

[48] Rahman I.A., Tsuboi K., Hussain Z., Yamashita R., Okamoto Y., Uyama T. (2016). Calcium-dependent generation of N-acylethanolamines and lysophosphatidic acids by glycerophosphodiesterase GDE7. Biochim. Biophys. Acta.

[49] Key C.C., Bishop A.C., Wang X., Zhao Q., Chen G.Y., Quinn M.A. (2020). Human GDPD3 overexpression promotes liver steatosis by increasing lysophosphatidic acid production and fatty acid uptake. J. Lipid Res..

[50] Naka K., Ochiai R., Matsubara E., Kondo C., Yang K.M., Hoshii T. (2020). The lysophospholipase D enzyme Gdpd3 is required to maintain chronic myelogenous leukaemia stem cells. Nat. Commun..

[51] Ohshima N., Kudo T., Yamashita Y., Mariggio S., Araki M., Honda A. (2015). New members of the mammalian glycerophosphodiester phosphodiesterase family: GDE4 and GDE7 produce lysophosphatidic acid by lysophospholipase D activity. J. Biol. Chem..

[52] Tserendavga B., Ohshima N., Fujita C., Yuzawa K., Ohshima M., Yanaka N. (2021). Characterization of recombinant murine GDE4 and GDE7, enzymes producing lysophosphatidic acid and/or cyclic phosphatidic acid. J. Biochem..

[53] Chang P.A., Shao H.B., Long D.X., Sun Q., Wu Y.J. (2008). Isolation, characterization and molecular 3D model of human GDE4, a novel membrane protein containing glycerophosphodiester phosphodiesterase domain. Mol. Membr. Biol..

[54] Bose J.L., Fey P.D., Bayles K.W. (2013). Genetic tools to enhance the study of gene function and regulation in *Staphylococcus aureus*. Appl. Environ. Microbiol..

[55] Okudaira M., Inoue A., Shuto A., Nakanaga K., Kano K., Makide K. (2014). Separation and quantification of 2-acyl-1-lysophospholipids and 1-acyl-2-lysophospholipids in biological samples by LC-MS/MS. J. Lipid Res..

[56] Huynh K., Partch C.L. (2015). Analysis of protein stability and ligand interactions by thermal shift assay. Curr. Protoc. Protein Sci..

[57] Zhao H., Ghirlando R., Alfonso C., Arisaka F., Attali I., Bain D.L. (2015). A multilaboratory comparison of calibration accuracy and the performance of external references in analytical ultracentrifugation. PLoS One.

[58] Schuck P. (2000). Size-distribution analysis of macromolecules by sedimentation velocity ultracentrifugation and lamm equation modeling. Biophys. J..

[59] Laue T.M., Shah B.D., Ridgeway T.M., Pelletier S.L. (1992).

[60] Otwinowski Z., Minor W. (1997). Processing of X-ray diffraction data collected in oscillation mode. Meth. Enzymol..

[61] Karplus P.A., Diederichs K. (2012). Linking crystallographic model and data quality. Science.

[62] McCoy A.J., Grosse-Kunstleve R.W., Adams P.D., Winn M.D., Storoni L.C., Read R.J. (2007). Phaser crystallographic software. J. Appl. Crystallogr..

[63] Tunyasuvunakool K., Adler J., Wu Z., Green T., Zielinski M., Zidek A. (2021). Highly accurate protein structure prediction for the human proteome. Nature.

[64] Pettersen E.F., Goddard T.D., Huang C.C., Meng E.C., Couch G.S., Croll T.I. (2021). UCSF ChimeraX: structure visualization for researchers, educators, and developers. Protein Sci..

[65] Mirdita M., Schutze K., Moriwaki Y., Heo L., Ovchinnikov S., Steinegger M. (2022). ColabFold: making protein folding accessible to all. Nat. Met..

[66] Afonine P.V., Grosse-Kunstleve R.W., Echols N., Headd J.J., Moriarty N.W., Mustyakimov M. (2012). Towards automated crystallographic structure refinement with phenix.refine. Acta Crystallogr. D Biol. Crystallogr..

[67] Emsley P., Lohkamp B., Scott W.G., Cowtan K. (2010). Features and development of coot. Acta Crystallogr. D Biol. Crystallogr..

