# Supplementary material for: Lysophosphatidylglycerol (LPG) phospholipase D maintains membrane homeostasis in Staphylococcus aureus by converting LPG to lysophosphatidic acid
Source: J Biol Chem. 2023 May 25;299(7):104863. doi: 10.1016/j.jbc.2023.104863 (PMC10404611; doi:10.1016/j.jbc.2023.104863)
Supplement: Supplementary material [file mmc1.pdf]

Supplementary Material

Lysophosphatidylglycerol (LPG) phospholipase D maintains membrane homeostasis in *Staphylococcus aureus* by converting LPG to lysophosphatidic acid

Chitra Subramanian, Mi-Kyung Yun, Matthew M. Frank and Charles O. Rock

Tables S1-S3
Figure S1-S10

Table S1
Strains and plasmids used in this work

Strain Name	Relevant Genotype	Description	Source
JE2	JE2 USA300	USA300 CA-MRSA strain LAC	Fey, et al.[a]
NE654	*SAUSA300_0604*::ϕNΣ	α/β hydrolase fold protein	Fey, et al.
NE960	*SAUSA300_0601*::ϕNΣ	α/β hydrolase fold protein	Fey, et al.
NE1611	*SAUSA300_1271*::ϕNΣ	α/β hydrolase fold protein	Fey, et al.
NE204	*SAUSA300_1194*::ϕNΣ	α/β hydrolase fold protein	Fey, et al.
NE1779	*SAUSA300_2518*::ϕNΣ	α/β hydrolase fold protein	Fey, et al.
NE28	*SAUSA300_0947*::ϕNΣ	α/β hydrolase fold protein	Fey, et al.
NE1534	*SAUSA300_0070*::ϕNΣ	putative lysophospholipase	Fey, et al.
NE1773	*SAUSA300_2457*::ϕNΣ	phospholipase/carboxyesterase	Fey, et al.
NE1775	*SAUSA300_0320*::ϕNΣ	Geh inactivation	Fey, et al.
NE104	*SAUSA300_0641*::ϕNΣ	SAL3 inactivation	Fey, et al.
NE212	*SAUSA300_1710*::ϕNΣ	putative lysophospholipase	Fey, et al.
NE338	*SAUSA300_2603*::φNΣ	Lip inactivation	Fey, et al.
NE503	*SAUSA300_2564*::ϕNΣ	EstA inactivation	Fey, et al.
NE639	*SAUSA300_0030*::ϕNΣ	GDPD protein	Fey, et al.
NE678	*SAUSA300_0099*::ϕNΣ	Plc	Fey, et al.
NE778	*SAUSA300_0862*::ϕNΣ	GlpQ	Fey, et al.
NE1544	*SAUSA300_1020*::ϕNΣ	LpgD	Fey, et al.
NE1122	*SAUSA300_0763*::ϕNΣ	Est	Fey, et al.
NE1661	*SAUSA300_1667*::ϕNΣ	GDPD protein	Fey, et al.
NE371	*SAUSA300_0222*::ϕNΣ	Putative membrane protein	Fey, et al.
AH1263	SAUSA300 (LAC)	USA300 CA-MRSA ErmS strain	Parsons, et al.[b]
PDJ73	AH1263 (Δ*lpgD*)	*lpgD* gene deletion	Present Study
**Plasmids**		**Description**	**Source**
pPJ654		pET28a expressing LpgDΔN	Present study
pCN51		Pcad promoter	Charpentier, et al.[c]
pPJ659		pCN51 expressing LpgD	Present study
pPJ660		pCN51 expressing LpgDΔN	Present study
pPJ663		pET28a expressing LpgDΔN(E78A)	Present study
pPJ661		pET28a expressing LpgDΔN(H51A)	Present study
pPJ664		pET28a expressing LpgDΔN(H93A)	Present study
pPJ662		pET28a expressing LpgDΔN(H51A,H93A)	Present study

[a] Fey PD, Endres JL, Yajjala VK, Widhelm TJ, Boissy RJ, Bose JL, Bayles KW A genetic resource for rapid and comprehensive phenotype screening of nonessential *Staphylococcus aureus* genes. *MBio* 2013;4:e00537-00512.

[b] Parsons, J. B., Broussard, T. C., Bose, J. L., Rosch, J. W., Jackson, P., Subramanian, C., and Rock, C. O. (2014) Identification of a two-component fatty acid kinase responsible for host fatty acid incorporation by *Staphylococcus aureus*. *Proc Natl Acad Sci U S A* **111**, 10532-10537

[c] Charpentier, E., Anton, A. I., Barry, P., Alfonso, B., Fang, Y., and Novick, R. P. (2004) Novel cassette-based shuttle vector system for gram-positive bacteria. *Appl. Environ. Microbiol.* **70**, 6076-6085

Table S2
Sedimentation velocity analysis of LpgDΔN.

Sample	μM [a]	s_{20} *(Svedberg)*[b]	$s_{20,w}$ *(Svedberg)*[c]	Mw (Da)[d]	f/f_0 [e]
LpgDΔN	14.08	2.62 (90%)	2.85	33,306 (33,430)	1.28
		4.09 (6%)	4.43	64,697	1.28
		1.88 (3%)	2.04	20,267	1.28

[a] Total concentration in micro-molar.

[b] Sedimentation coefficient taken from the ordinate maximum of each peak in the best-fit *c(s)* distribution at 20 °C with percentage protein amount in parenthesis. Sedimentation coefficient (*s*-value) is a measure of the size and shape of a protein in a solution with a specific density and viscosity at a specific temperature.

[c] Standard sedimentation coefficient ($s_{20,w}$ -value) in water at 20 °C.

[d] Molar mass values (MW) taken from the *c(s)*-transformed *c(M)* distribution. Theoretical MW of the monomer in parenthesis.

[e] Best-fit weight-average frictional ratio values $(f/f_0)_w$ taken from the *c(s)* distribution.

Table S3
Data collection and refinement statistics for LpgDΔN structures.

Parameter	PDB ID: 8GHH	PDB ID: 8GHI
**Data Collection**[a]		
Wavelength (Å)	1.0	1.0
Space group	I422	I422
Cell dimensions		
a (Å)	108.0	108.0
b (Å)	108.0	108.0
c (Å)	138.4	139.7
α (°)	90.0	90.0
β (°)	90.0	90.0
γ (°)	90.0	90.0
Resolution range (Å)	42.56-2.10 (2.15-2.10)	45.66-2.40 (2.45-2.40)
R_{merge}	0.079 (0.881)	0.119 (1.041)
CC1/2	0.999 (0.894)	0.989 (0.447)
Completeness (%)	100.0 (99.7)	95.7 (99.7)
Redundancy	28.6 (20.9)	4.7 (5.4)
Mean $I/\sigma(I)$	26.2 (2.8)	7.4 (1.1)
Unique reflections	24,165 (1,490)	15,817 (1,014)
**Refinement**[b]		
Resolution range (Å)	42.56-2.10	38.20-2.40
No. of reflections	24,162	15,813
No. of atoms		
Protein	2,205	2,179
Ligand	53	11
Water	84	16
R_{work}	0.188	0.225
R_{free}	0.215	0.249
Average B factors ($Å^2$)		
Protein	59.0	83.6
Ligand	79.7	98.4
Water	59.4	79.1
Rmsd from ideal values		
Bond lengths (Å)	0.003	0.002
Bond angles (˚)	0.604	0.468
Ramachandran plot		
Favored (%)	99.3	99.3
Allowed (%)	0.7	0.7
Outliers (%)	0.0	0.0

[a] Values in parentheses refer to the highest resolution shell. Statistics for data collection were calculated using Xtriage.
[b] Refinement statistics were obtained by PHENIX refinement.

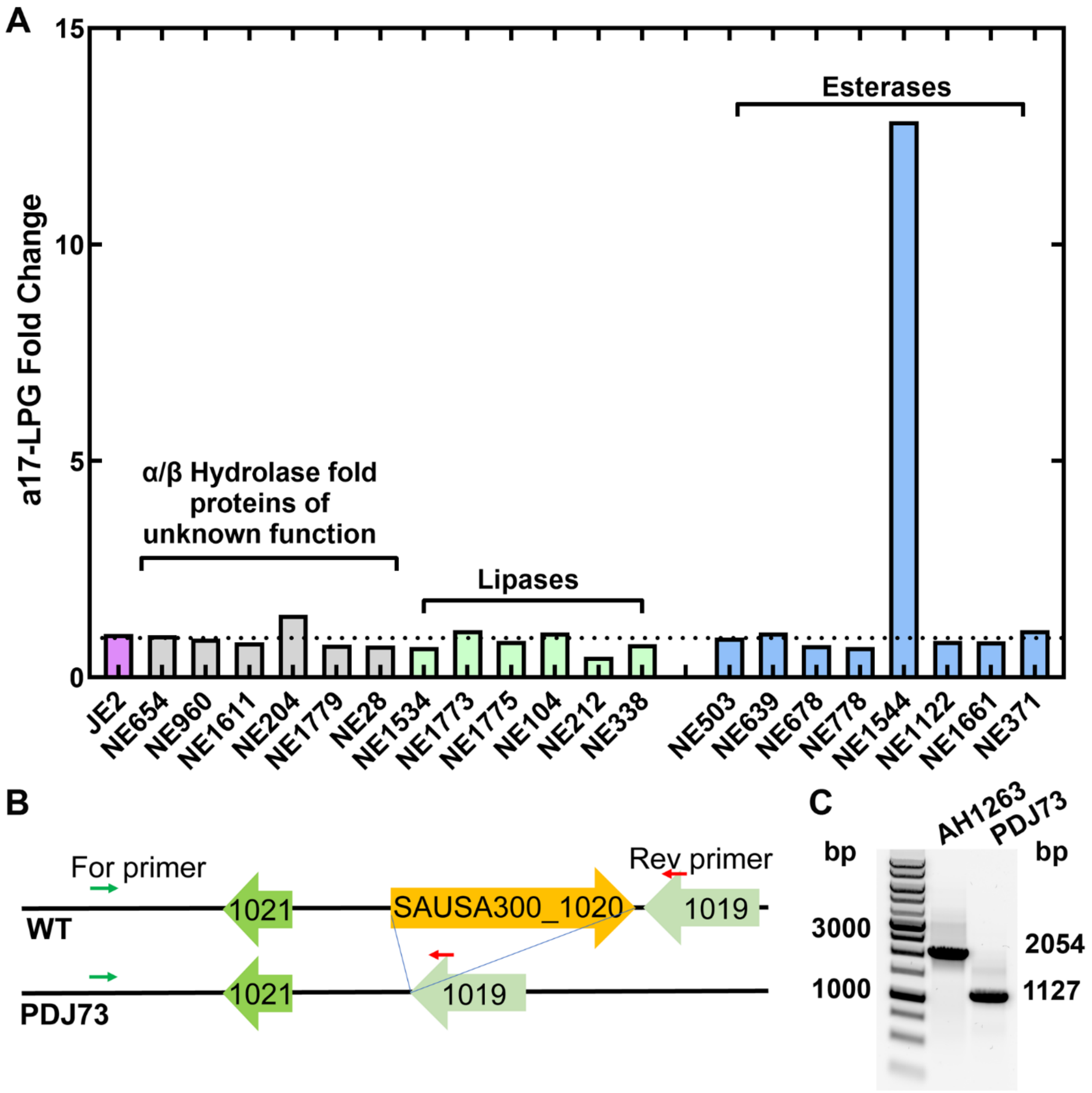

Figure S1. A screen of knockout strains in genes potentially involved in LPG metabolism. *A*, a panel of mutant strains from the Nebraska transposon library (Table S1) were grown to an A_{600} of 4.0, and the fold differences in the cellular levels of a17-LPG in each strain were compared to wild-type strain JE2 by LC-MS/MS using [d5]17-LPG as the internal standard. *B*, schematic diagram for the deletion and analysis of *lpgD* from the AH1263 genome. *C*, an agarose gel showing PCR confirmation of the genetic organization in strain AH1263 (wild-type) and strain PDJ73 (Δ*lpgD*).

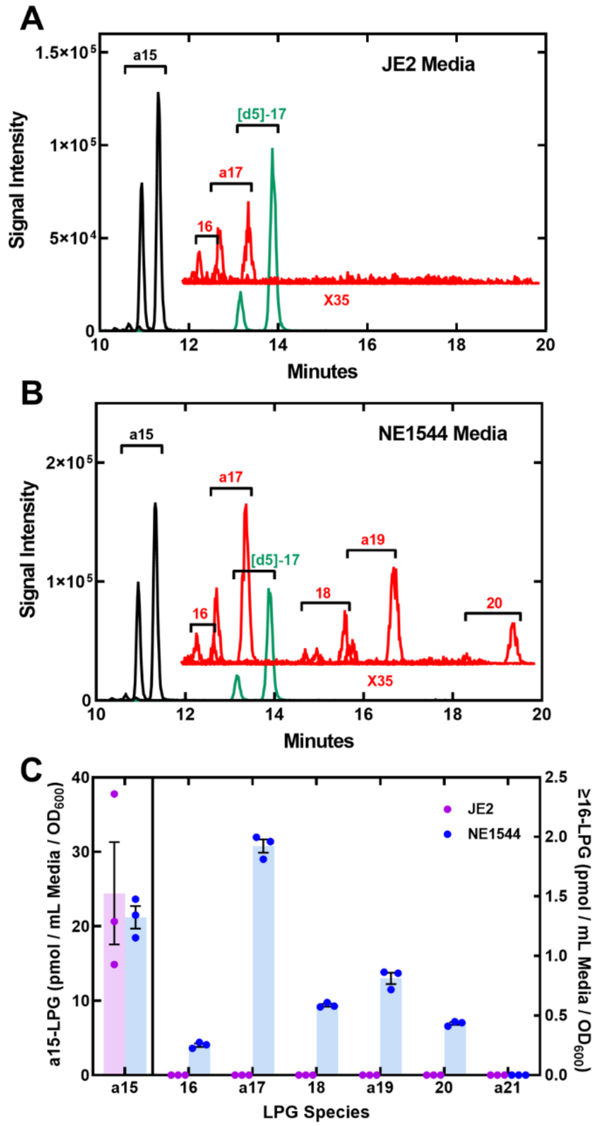

Figure S2. Size and composition of the extracellular LPG pool in strains JE2 (wild-type) and NE1544 (*lpgD*::ϕNΣ). *A*, a representative LC-MS/MS scan of LPG molecular species found in the media supernatant of wild-type strain JE2 with the [d5]17-LPG internal standard (green), a15-LPG (black) and ≥16-LPG (red). *Inset*, the red ≥16-LPG trace is increased by 35-fold to illustrate the distribution of ≥16-LPG present. *B*, a representative LC-MS/MS scan of LPG molecular species found in the media supernatant of strain NE1544 (*lpgD*::ϕNΣ) with the [d5]17-LPG internal standard (green), a15-LPG (black) and ≥16-LPG (red). *Inset*, the red ≥16-LPG trace is increased by 35-fold to illustrate the distribution of ≥16-LPG present. *C*, quantitation of the media concentrations of LPG molecular species in strains JE2 (wild-type) and NE1544 (*lpgD*::ϕNΣ) determined by LC-MS/MS using [d5]17-LPG as the internal standard.

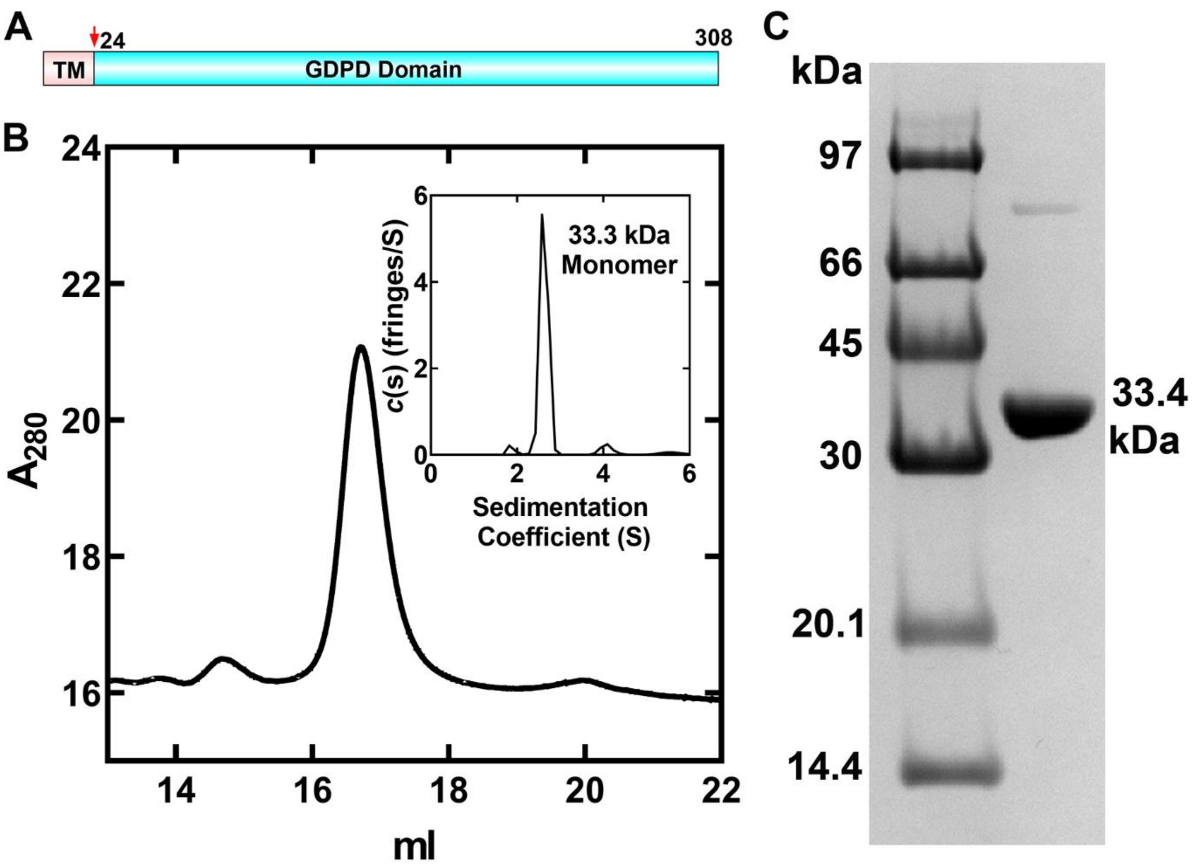

Figure S3. Sequence analysis and purification of LpgD. *A*, the LpgD protein has a predicted amino terminal transmembrane helix connected to a glycerophosphodiester phosphodiesterase (GDPD) domain. *B*, the GDPD domain was expressed as an amino-terminal His-tagged protein (LpgDΔN) lacking amino acids 1-24, purified by affinity chromatography and analyzed using a Superdex 200 gel filtration column. *Inset*, the protein was a monomer of 33.3 kDa based on analytical ultracentrifugation analysis of LpgDΔN. *C*, SDS gel electrophoresis showing LpgDΔN purity.

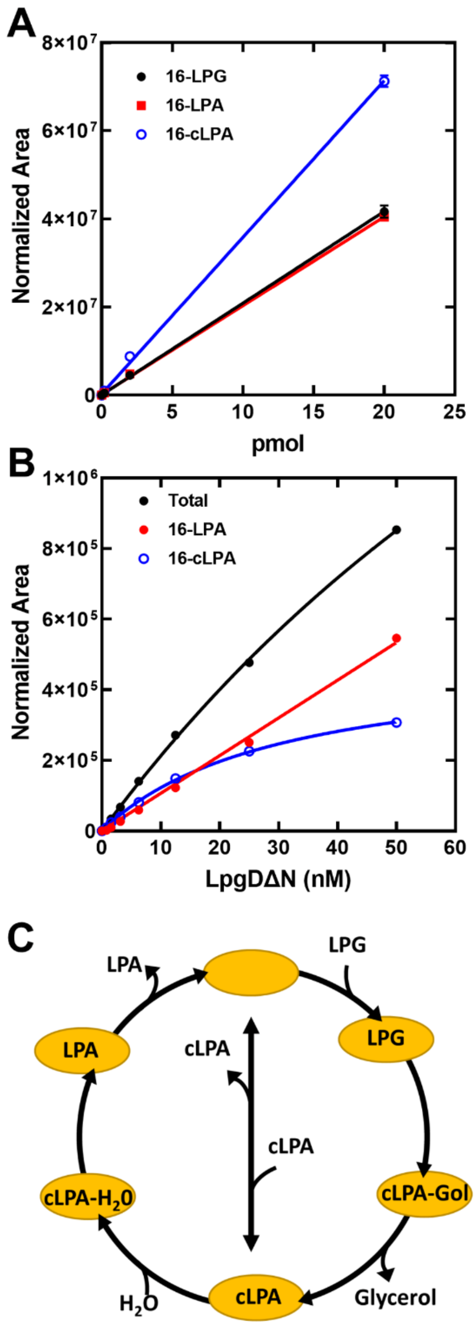

Figure S4. MS standard curves, LpgDΔN protein curve and the LpgD catalytic cycle. *A*, the standard curves for LPG, LPA and cLPA determined by LC-MS/MS. The equations were: 16-LPG, y = 416,262x + 53,339; 16-LPA, y = 403,292x + 126,440; 16-cLPA, y = 710,749x + 268,550. *B*, a representative LpgDΔN protein curve measuring the formation of LPA, cLPA and the total amount of product. *C*, LpgD (yellow oval) carries out an two-step reaction. First, LPG binds to LpgD and cLPA is formed. Glycerol (Gol) then dissociates from the enzyme allowing water to bind. cLPA is then broken down to LPA, which is released from LpgD to complete the cycle. Some cLPA dissociates from the enzyme following glycerol release and can re-bind free LpgD to convert cLPA to LPA.

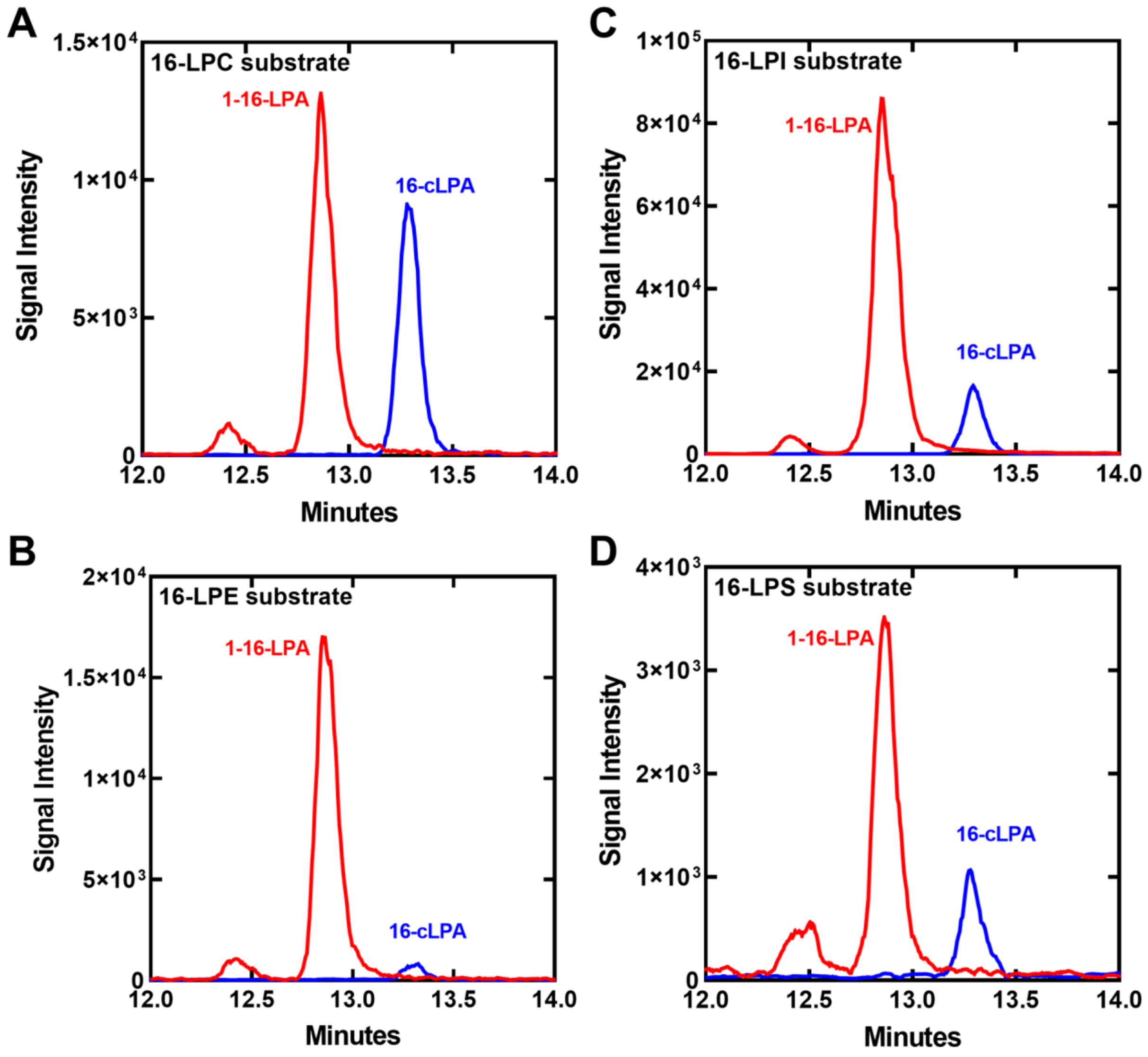

Figure S5. Headgroup selectivity of LpgDΔN. The lysophospholipid substrates were compared using 20 nM LpgDΔN, 250 μM Mn^{2+} and 30 μM of each substrate. The LPA (red) and cLPA (blue) LpgDΔN products were separated by LC-MS/MS. *A*, products of LpgDΔN digestion of 16-LPC. *B*, products of LpgDΔN digestion of 16-LPE. *C*, products of LpgDΔN digestion of 16-LPI. *D*, products of LpgDΔN digestion of 16-LPS

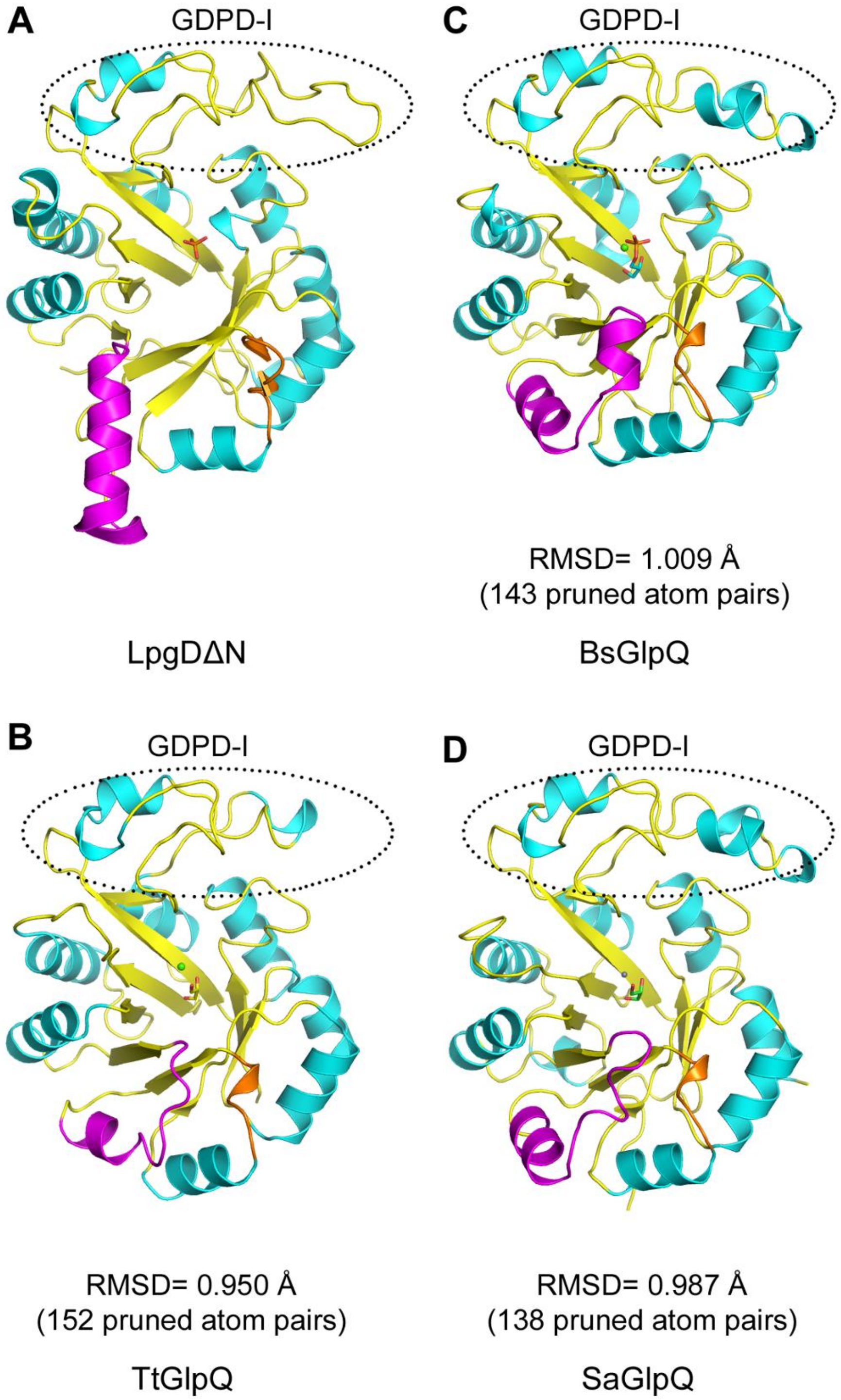

Figure S6. Comparison between LpgDΔN and three representative GlpQ (GDPD) proteins. The crystal structure of LpgDΔN is compared to three GlpQ structures. The root mean square deviations (RMSD) between LpgDΔN and the other compared structures were calculated using the MatchMaker program within ChimeraX. The GDPD insert region (GDPD-I) is highlighted with the dashed oval. *A*, LpgDΔN (PDB ID: 8GHH). *B*, *T. tengcongensis* GlpQ (PDB ID: 2PZ0). *C*, *B. subtilis* GlpQ (PDB ID: 5T9C). *D*, *S. aureus* GlpQ (PDB ID: 2OOG). Helices are cyan, sheets and loops are yellow, helix α6 is magenta, and loop L7 is orange. Active site ligands are shown as sticks.

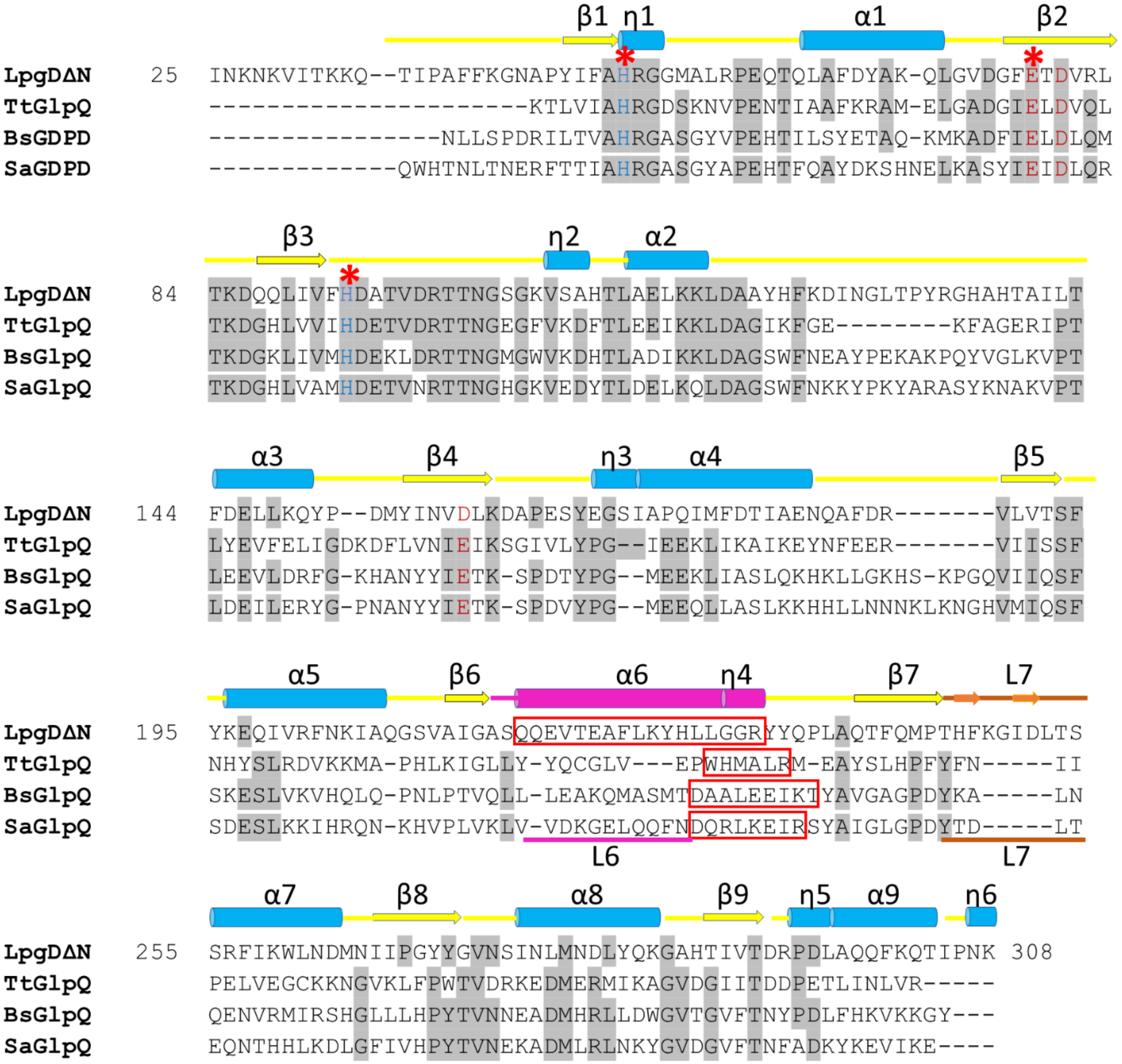

Figure S7. Multiple sequence alignment of LpgDΔN and three prototypical GDPD (GlpQ) proteins. The structure-based sequence alignment of LpgDΔN (PDB ID 8GHH), *T. tengcongensis* GlpQ (PDB ID: 2PZ0), *B. subtilis* GlpQ (PDB ID: 5T9C), and *S. aureus* GlpQ (PDB ID: 2OOG) was performed with Chimera and adjusted manually using PyMOL. Secondary structure of LpgDΔN is defined by the DSSP algorithm in PyMOL and the secondary structural elements of LpgDΔN are depicted above the sequence alignment. α-Helices and β-strands are colored cyan and yellow, respectively. Loop L6 and helix α6 are colored magenta and loop L7 is colored orange (see Fig. 4). The helices α6 are outlined by a red box. Light grey regions show residues conserved in at least three sequences. The catalytic histidine and metal binding residues are colored blue and red, respectively. Residues mutated in LpgDΔN are indicated with red asterisks.

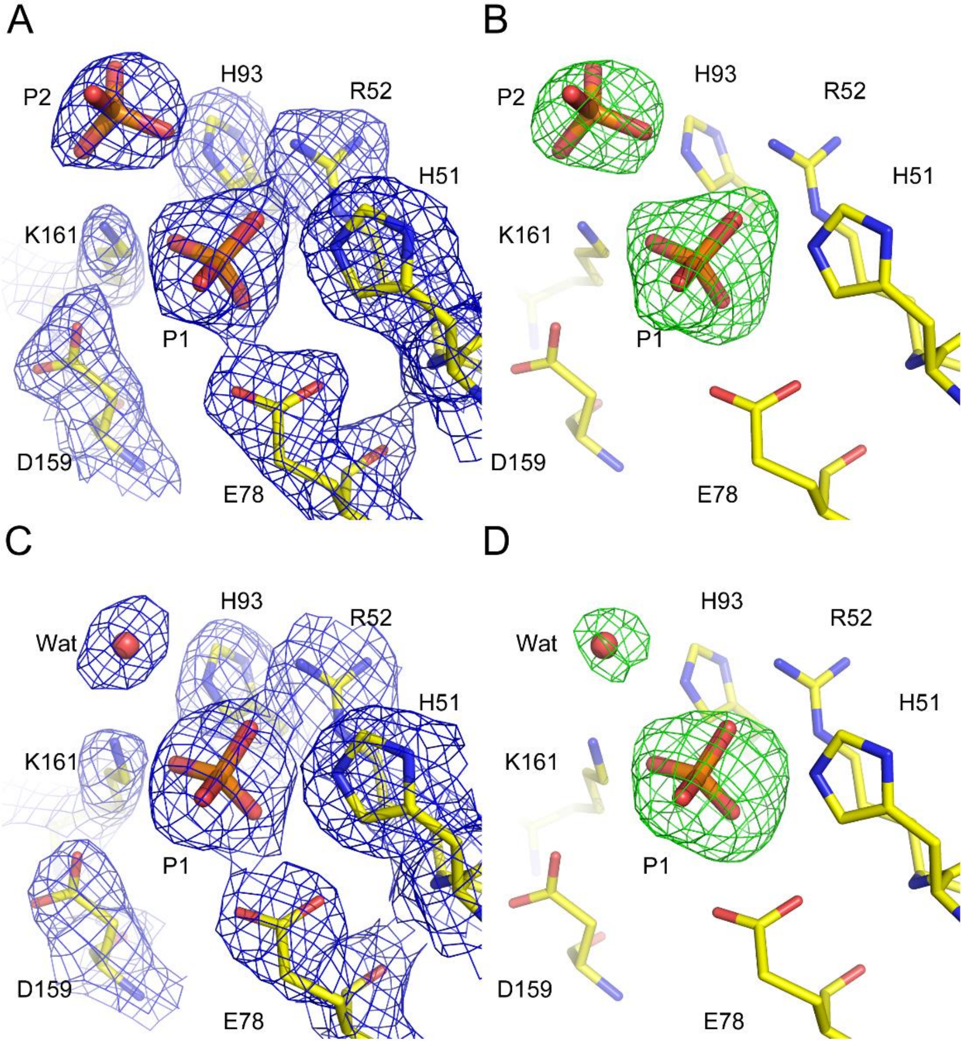

Figure S8. Electron density maps of the LpgD active site. *A*, the 2Fo-Fc electron density map (blue mesh) of the LpgDΔN active site in crystals obtained at pH 5.3 (PDB ID: 8GHH). Two dihydrogen phosphate ions, P1 and P2, are modelled in the observed density. *B*, the Fo-Fc simulated annealing omit map generated from the refined LpgDΔN structure at pH 5 (PDB ID: 8GHH) with both P1 and P2 omitted. *C*, the 2Fo-Fc electron density map (blue mesh) of the LpgDΔN active site observed in crystals soaked with pH 6.5 buffer (PDB ID: 8GHI). *D*, the Fo-Fc simulated annealing omit map generated from the refined LpgDΔN structure at pH 6.5 (PDB ID: 8GHI) with both P1 and water molecule omitted. The 2Fo-Fc electron density maps (blue mesh) and Fo-Fc simulated annealing omit maps (green mesh) are contoured at 1 σ and 3 σ, respectively.

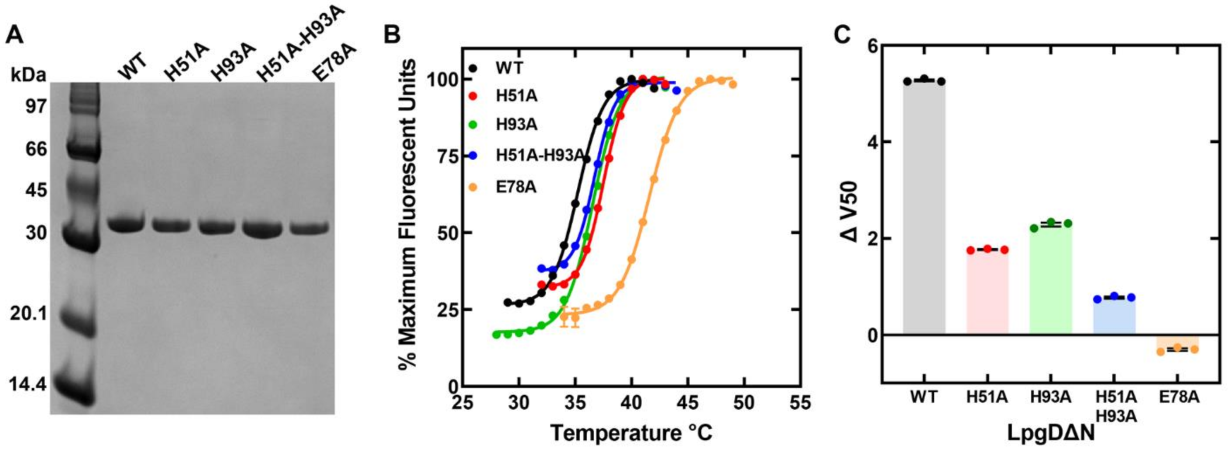

Figure S9. Purity and thermal stabilities of the LpgDΔN mutants. LpgDΔN and four additional mutant proteins; LpgDΔN(H51A), LpgDΔN(H93A), LpgDΔN(H51A, H93A) and LpgDΔN(E78A) were expressed and purified by affinity and gel filtration chromatography. *A*, SDS gel electrophoresis illustrating the purity of LpgDΔN and its four mutant derivatives. *B*, thermal stabilities of LpgDΔN and its mutant derivatives. *C*, Mn^{2+}-induced shift in the thermal stabilities of the mutant protein panel.

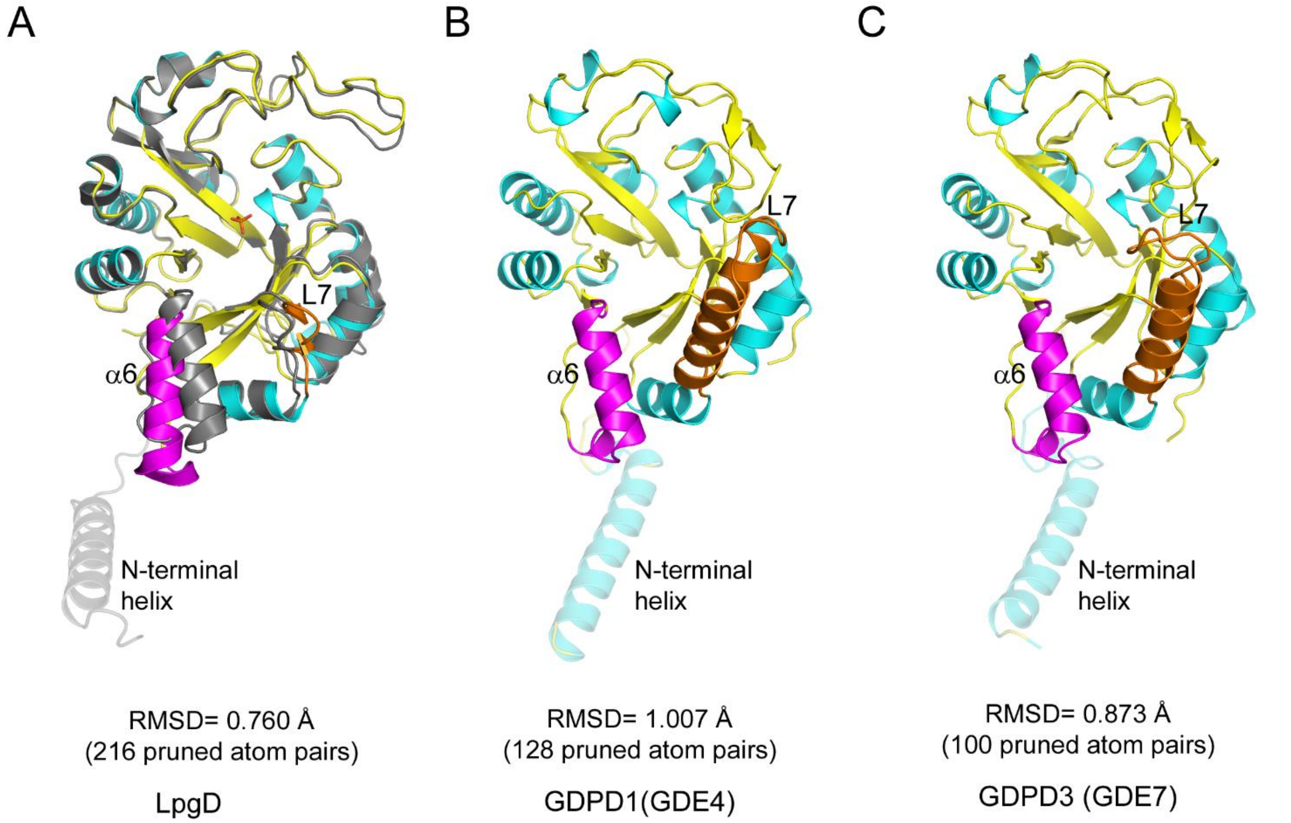

Figure S10. Comparison between LpgDΔN and two related human GDPD proteins. *A*, a comparison of the AlphaFold model of LpgD (AlphaFold model ID: Q2FZF9) depicted in grey to the determined crystal structure of LpgDΔN (PDB ID: 8GHH). *B*, the predicted human GDPD1 (GDE4) (AlphaFold model ID: Q8N9F7). *C*, the predicted structure of human GDPD3 (GDE7) (AlphaFold model ID: Q7L5L3). Helices are cyan, sheets and loops are yellow, helix α6 is magenta, and loop L7 is orange. The root mean square deviations (RMSD) between the crystal structure of LpgDΔN and the compared AlphaFold structures were calculated using MatchMaker within ChimeraX.
